# Supplementary material for: Consumer acceptance of personalised nutrition: The role of ambivalent feelings and eating context
Source: PLoS One. 2020 Apr 13;15(4):e0231342. doi: 10.1371/journal.pone.0231342 (PMC7153894; doi:10.1371/journal.pone.0231342)
Supplement: S1 Data — Original questionnaire (in Dutch). (PDF) [file pone.0231342.s001.pdf]

PERSONALISED NUTRITION - ORIGINAL DUTCH QUESTIONNAIRE

Welkom bij dit onderzoek!

Dit onderzoek wordt uitgevoerd in opdracht van Wageningen Universiteit & Research als onderdeel van een groot onderzoeksconsortium**. In deze studie zijn wij geïnteresseerd in uw keuzes en meningen over gepersonaliseerd voedingsadvies**. Deelname is vrijwillig en uw antwoorden worden anoniem behandeld. Alleen de partners in het onderzoeksconsortium kunnen toegang krijgen tot de (geanonimiseerde) data.

Instructie

- Bij de vragen worden alle mogelijke antwoorden weergegeven. Kruis steeds het hokje aan van het antwoord dat het meest op u van toepassing is.
- Het is de bedoeling dat u slechts één hokje aankruist. Indien meerdere antwoorden kunnen worden gegeven, staat dat bij de vraag aangegeven.
- Diverse keren wordt gevraagd aan te geven in hoeverre u het (on)eens bent met een aantal uitspraken. Als de stelling helemaal niet kenmerkend is voor u, vink dan een "1" aan; als de stelling helemaal wel kenmerkend is voor u, vink dan een "7" aan. En gebruik de cijfers in het midden als uw antwoord tussen deze twee extremen valt.
- Deze vragenlijst duurt ongeveer 20 minuten.

**NB: Het kan voorkomen dat er vragen zijn die u moeilijk kunt beantwoorden. Probeer dan toch een antwoord te geven. Er zijn geen goede of foute antwoorden, het gaat om uw (eerste) indruk.**

Klik verder als u akkoord bent met deelname aan het onderzoek.

[PAGE BREAK]

Onderdeel 1. Gepersonaliseerd voedingsadvies

Deze vragenlijst gaat over gepersonaliseerd voedingsadvies. Gepersonaliseerd voedingsadvies is advies dat volledig afgestemd is op u. Er kan op verschillende manieren gepersonaliseerd worden:

- persoonlijke voorkeuren (bijv. wat vindt u wel en niet lekker),
- zelf gestelde doelen/wensen (bijv. meer fruit eten of verlagen van cholesterol),
- eetgewoonten,
- gezondheidssituatie (bijv. overgewicht, bloeddruk en cholesterol),
- en zelfs DNA profiel.

ICT-ontwikkelingen spelen hierop in met 'gezondheidstrackers', zoals smartwatches, stappentellers en smartphone apps, waarmee u inzicht krijgt in uw eigen gedrag of gezondheid.

Een voorbeeld:
- Algemeen voedingsadvies: het Voedingscentrum adviseert iedereen om dagelijks een x aantal volkoren boterhammen te eten.
- Gepersonaliseerd voedingsadvies: op basis van uw persoonlijke voorkeur en gezondheidsstatus (bijv. extra behoefte aan eiwit) kunnen wij u bijvoorbeeld adviseren om een bakje yoghurt met havermout te eten in plaats van brood, omdat dat beter bij u past.

Met de volgende vragenlijst willen we te weten komen hoe u denkt over gepersonaliseerd voedingsadvies.

[PAGE BREAK]

1. Understanding definition question[1]

ITEMS RANDOM	Helemaal niet 1	2	Neutraal 3	4	5	6	Helemaal wel 7
Heeft u een duidelijk beeld van wat gepersonaliseerd voedingsadvies inhoudt?	☐	☐	☐	☐	☐	☐	☐

[PAGE BREAK]

2. Intention to make use of personalised nutrition advice

Geef aan of u het eens of oneens bent met de volgende uitspraken:

ITEMS RANDOM	Helemaal mee oneens 1	2	Neutraal 3	4	5	6	Helemaal mee eens 7
1. Ik ben van plan gepersonaliseerd voedingsadvies te gaan gebruiken.	☐	☐	☐	☐	☐	☐	☐
2. Ik zou het gebruik van gepersonaliseerd voedingsadvies overwegen.	☐	☐	☐	☐	☐	☐	☐
3. Ik ga absoluut gebruik maken van gepersonaliseerd voedingsadvies.	☐	☐	☐	☐	☐	☐	☐

[PAGE BREAK]

[1] These headings are added for the researchers (so that they know what construct/ concept the specific question belongs to), but were not shown to the participants.

3. Ambivalent Feelings

Gepersonaliseerd voedingsadvies.... ITEMS RANDOM	1	2	3	4	5	6	7	
roept bij mij absoluut geen tegenstrijdige gevoelens op	☐	☐	☐	☐	☐	☐	☐	roept bij mij zeer veel tegenstrijdige gevoelens op
geeft mij absoluut geen ongemakkelijk gevoel	☐	☐	☐	☐	☐	☐	☐	geeft mij een zeer ongemakkelijk gevoel
roept bij mij geen gemengde gevoelens op	☐	☐	☐	☐	☐	☐	☐	roept bij mij sterke gemengde gevoelens op

[PAGE BREAK]

4. Benefits & risks personalised nutrition

Items 1-3: Personalisation Benefit
Items 4-6: Service Effectiveness > *NB. Not used as variable in the study reported in the manuscript*
Items 7-9: Privacy Risk

Vergeleken met algemeen voedingsadvies, is gepersonaliseerd voedingsadvies: ITEMS RANDOM	Helemaal mee oneens 1	2	3	Neutraal 4	5	6	Helemaal mee eens 7
1. Beter afgestemd op mijn gezondheidsbehoeften.	☐	☐	☐	☐	☐	☐	☐
2. Relevanter voor mijn gezondheid.	☐	☐	☐	☐	☐	☐	☐
3. Beter voor mijn gezondheid.	☐	☐	☐	☐	☐	☐	☐
4. Een advies waarmee ik een gezondere levensstijl kan ontwikkelen.	☐	☐	☐	☐	☐	☐	☐
5. Een advies dat mij helpt om gezond te leven.	☐	☐	☐	☐	☐	☐	☐
6. Een advies dat mij het gevoel geeft controle te hebben over het ontwikkelen van een gezondere levensstijl.	☐	☐	☐	☐	☐	☐	☐
7. Een advies dat veel privacy-gerelateerde risico's met zich meebrengt.	☐	☐	☐	☐	☐	☐	☐
8. Een gevaar voor mijn privacy.	☐	☐	☐	☐	☐	☐	☐
9. Een groot risico voor mijn privacy.	☐	☐	☐	☐	☐	☐	☐

[PAGE BREAK]

5. Perceived benefits

NB. Not used as variable in the study reported in the manuscript

Welke van de volgende voordelen zou gepersonaliseerd voedingsadvies aantrekkelijk voor u maken? ITEMS RANDOM	Helemaal mee oneens 1	2	3	Neutraal 4	5	6	Helemaal mee eens 7
1. Weten welk voedsel het beste is voor uw gezondheid	☐	☐	☐	☐	☐	☐	☐
2. Gewichtsafname	☐	☐	☐	☐	☐	☐	☐
3. Gewichtstoename	☐	☐	☐	☐	☐	☐	☐
4. Fitheid	☐	☐	☐	☐	☐	☐	☐
5. Het verbeteren van de gezondheid van mijn gezin	☐	☐	☐	☐	☐	☐	☐
6. Het verbeteren van mijn gezondheid	☐	☐	☐	☐	☐	☐	☐
7. Het verbeteren van mijn kwaliteit van leven	☐	☐	☐	☐	☐	☐	☐
8. Het verbeteren van sportprestaties	☐	☐	☐	☐	☐	☐	☐
9. Het opbouwen van een gezonde huid	☐	☐	☐	☐	☐	☐	☐
10. Het voorkomen van een toekomstige ziekte	☐	☐	☐	☐	☐	☐	☐
11. Het voorkomen van een erfelijke ziekte	☐	☐	☐	☐	☐	☐	☐

[PAGE BREAK]

6. Barriers personalised nutrition (1) – data protection

NB. Not used as variable in the study reported in the manuscript

Geef aan in hoeverre u het eens of oneens bent met de volgende uitspraken:<br>ITEMS RANDOM	Helemaal mee oneens<br>1	2	3	Neutraal<br>4	5	6	Helemaal mee eens<br>7
1. Ik ben bang dat een gepersonaliseerd voedingsadvies niet effectief is.	☐	☐	☐	☐	☐	☐	☐
2. Ik ben bezorgd over de manier waarop autoriteiten om zouden kunnen gaan met mijn persoonlijke gegevens.	☐	☐	☐	☐	☐	☐	☐
3. Ik ben bang dat mijn persoonlijke gegevens niet vertrouwelijk worden behandeld.	☐	☐	☐	☐	☐	☐	☐
4. Ik ben bezorgd over hoe mijn persoonlijke gegevens en testresultaten opgeslagen worden.	☐	☐	☐	☐	☐	☐	☐
5. Ik ben bezorgd over hoe mijn persoonlijke gegevens gebruikt zouden kunnen worden door verstrekkers van gepersonaliseerd voedingsadvies.	☐	☐	☐	☐	☐	☐	☐
6. Ik ben bezorgd over hoe mijn persoonlijke gegevens gebruikt zouden kunnen worden door adverteerders.	☐	☐	☐	☐	☐	☐	☐
7. Ik ben bezorgd over hoe mijn persoonlijke gegevens gebruikt zouden kunnen worden door verzekeringsmaatschappijen.	☐	☐	☐	☐	☐	☐	☐
8. Ik ben bang dat mijn persoonlijke gegevens door hackers bemachtigd zou kunnen worden.	☐	☐	☐	☐	☐	☐	☐

[PAGE BREAK]

7. Barriers personalised nutrition (2)

Items 1-5: Eating context barrier
Items 6-7: Liking of food barrier > *NB. Not used as variable in the study reported in the manuscript*
Items 8-10: Social context barrier > *NB. Not used as variable in the study reported in the manuscript*

Wat houdt u tegen om gepersonaliseerd voedingsadvies te gebruiken?<br>ITEMS RANDOM	Helemaal mee oneens<br>1	2	3	Neutraal<br>4	5	6	Helemaal mee eens<br>7
1. Dat iedereen in mijn gezin een gepersonaliseerd advies krijgt en daardoor iets anders zou moeten eten.	☐	☐	☐	☐	☐	☐	☐
2. Het is moeilijk om een gepersonaliseerd voedingsadvies op te volgen bij het eten in restaurants.	☐	☐	☐	☐	☐	☐	☐
3. Het is moeilijk om een gepersonaliseerd voedingsadvies op te volgen bij het eten bij andere mensen thuis.	☐	☐	☐	☐	☐	☐	☐
4. Het is moeilijk om een gepersonaliseerd voedingsadvies op te volgen tijdens het reizen.	☐	☐	☐	☐	☐	☐	☐
5. Het is moeilijk om een gepersonaliseerd voedingsadvies op te volgen op het werk.	☐	☐	☐	☐	☐	☐	☐
6. Eten aanbevolen krijgen dat ik niet lekker vind.	☐	☐	☐	☐	☐	☐	☐
7. Eten dat ik lekker vindt niet aanbevolen krijgen.	☐	☐	☐	☐	☐	☐	☐
8. Afwijzing van gepersonaliseerd voedingsadvies door mijn gezin.	☐	☐	☐	☐	☐	☐	☐
9. Afwijzing van gepersonaliseerd voedingsadvies door mijn vrienden.	☐	☐	☐	☐	☐	☐	☐
10. Afwijzing van gepersonaliseerd voedingsadvies door de samenleving.	☐	☐	☐	☐	☐	☐	☐

[PAGE BREAK]

8. *Trust in agencies to provide information on personalised nutrition*

NB. Not used as variable in the study reported in the manuscript

In hoeverre vertrouwt u de volgende verstrekkers van gepersonaliseerd advies?	Helemaal mee oneens			Neutraal			Helemaal mee eens
ITEMS RANDOM	1	2	3	4	5	6	7
1. Supermarkten	☐	☐	☐	☐	☐	☐	☐
2. Voedselfabrikanten	☐	☐	☐	☐	☐	☐	☐
3. Commerciële aanbieders van persoonlijk voedingsadvies	☐	☐	☐	☐	☐	☐	☐
4. Commerciële aanbieders van technologie	☐	☐	☐	☐	☐	☐	☐
5. Populaire diëten (Sonja Bakker, Voedselzandloper)	☐	☐	☐	☐	☐	☐	☐
6. Universiteiten	☐	☐	☐	☐	☐	☐	☐
7. Diëtisten	☐	☐	☐	☐	☐	☐	☐
8. Personal trainer / sportschool	☐	☐	☐	☐	☐	☐	☐
9. Huisarts	☐	☐	☐	☐	☐	☐	☐
10. Ziekenhuizen	☐	☐	☐	☐	☐	☐	☐
11. GGD (Gemeentelijke Gezondheidsdienst)	☐	☐	☐	☐	☐	☐	☐
12. Ministerie van Volksgezondheid	☐	☐	☐	☐	☐	☐	☐
13. Voedingscentrum	☐	☐	☐	☐	☐	☐	☐
14. Rijksinstituut voor Volksgezondheid en Milieu (RIVM)	☐	☐	☐	☐	☐	☐	☐
15. Vrienden	☐	☐	☐	☐	☐	☐	☐
16. Familie	☐	☐	☐	☐	☐	☐	☐
17. Mijn werkgever	☐	☐	☐	☐	☐	☐	☐

[PAGE BREAK]

9. *Risk-Benefit Calculus*

	Meer risico's			Neutraal			Meer voordelen
ITEMS RANDOM	1	2	3	4	5	6	7
Denkt u dat het gebruik van gepersonaliseerd voedingsadvies meer voordelen dan risico's biedt, of meer risico's dan voordelen?	☐	☐	☐	☐	☐	☐	☐

[PAGE BREAK]

Onderdeel 2. Achtergrondkenmerken

10. Intention to eat healthy/ stay eating healthy

NB. Not used as variable in the study reported in the manuscript

Geef aan of u het eens of oneens bent met de volgende uitspraken:<br>ITEMS RANDOM	Helemaal oneens<br>1	2	3	Neutraal<br>4	5	6	Helemaal eens<br>7
1. Ik ben van plan gezond te gaan/blijven eten.	☐	☐	☐	☐	☐	☐	☐
2. Ik zou overwegen om gezond te gaan/blijven eten.	☐	☐	☐	☐	☐	☐	☐
3. Ik ga/blijf absoluut gezond eten.	☐	☐	☐	☐	☐	☐	☐

[PAGE BREAK]

11. Subjective knowledge

NB. Not used as variable in the study reported in the manuscript

Geef aan in hoeverre de volgende uitspraken van toepassing zijn op u:<br>ITEMS RANDOM	Helemaal niet<br>1	2	3	Neutraal<br>4	5	6	Helemaal wel<br>7
1. Ik weet redelijk veel over gezonde voeding	☐	☐	☐	☐	☐	☐	☐
2. In mijn vriendenkring ben ik een van de 'experts' op het gebied van gezonde voeding	☐	☐	☐	☐	☐	☐	☐

[PAGE BREAK]

12. Self-efficacy

NB. Not used as variable in the study reported in the manuscript

	Heel moeilijk<br>1	2	3	Neutraal<br>4	5	6	Heel makkelijk<br>7
Hoe makkelijk zou het voor u zijn om gezonder te eten, als u dat echt zou willen?	☐	☐	☐	☐	☐	☐	☐

[PAGE BREAK]

13. Lifestyle

NB. Not used as variable in the study reported in the manuscript

Volgt u een bepaalde leefregel bij het eten? Meerdere antwoorden mogelijk.

☐ Nee
☐ Ja, ik ben flexitariër (af en toe vlees)
☐ Ja, ik ben vegetariër (geen vlees)
☐ Ja, ik ben veganist (helemaal geen dierlijke producten)
☐ Ja, vanwege mijn geloof
☐ Anders, namelijk.......................

[PAGE BREAK]

14. Food-related health problem

NB. Not used as variable in the study reported in the manuscript

Heeft u een voedingsgerelateerd gezondheidsprobleem? Meerdere antwoorden mogelijk

- ☐ Nee
- ☐ Ja, namelijk diabetes
- ☐ Ja, namelijk hoge bloeddruk
- ☐ Ja, namelijk overgewicht
- ☐ Ja, namelijk leverproblemen
- ☐ Ja, namelijk obstipatie
- ☐ Ja, namelijk verhoogd cholesterol
- ☐ Anders, namelijk...

[PAGE BREAK]

15. Diet

NB. Not used as variable in the study reported in the manuscript

Volgde u de afgelopen maand een dieet? Meerdere antwoorden mogelijk

- ☐ Nee
- ☐ Ja, een energiebeperkt dieet (vermageringsdieet)
- ☐ Ja, een natriumbeperkt dieet (zoutarm dieet)
- ☐ Ja, een vetbeperkt dieet
- ☐ Ja, een vezelrijk dieet
- ☐ Ja, een dieet bij diabetes mellitus (suikerziekte)
- ☐ Ja, een dieet bij hoog cholesterolgehalte
- ☐ Anders, namelijk.......................

[PAGE BREAK]

16. Subjective health

NB. Not used as variable in the study reported in the manuscript

Geef aan in hoeverre de volgende uitspraken van toepassing zijn op u:<br>ITEMS RANDOM	Heel ongezond<br>1	2	3	Neutraal<br>4	5	Heel gezond<br>6	7
1. Hoe gezond vindt u uzelf?	☐	☐	☐	☐	☐	☐	☐
2. Hoe gezond vindt u uw dieet in het algemeen (denk aan het laatste jaar)?	☐	☐	☐	☐	☐	☐	☐

[PAGE BREAK]

17.BMI

NB. Not used as variable in the study reported in the manuscript

Wat is uw lengte in centimeters?

...

Wat is uw gewicht in kilogram?

...

[PAGE BREAK]

18. Shopping

NB. Not used as variable in the study reported in the manuscript

De volgende vragen gaan over de boodschappen. ITEMS RANDOM	Nooit 1	Heel soms 2	Af en toe 3	Regel matig 4	Altijd 5
1. Hoe vaak doet u de boodschappen?	☐	☐	☐	☐	☐
2. Hoe vaak doet u online boodschappen?	☐	☐	☐	☐	☐

[PAGE BREAK]

19. Using personalised nutrition services

NB. Used as a selection question in the study: participants who indicated that they already use some form of personalised nutrition were omitted from the analyses, to avoid a bias in the results by people that already use personalised nutrition advice who may see benefits and risks differently based on their experiences.

Maakt u gebruik van gepersonaliseerde diensten?

☐ Nee
☐ Ja, ik ontvang gepersonaliseerd voedingsadvies
☐ Ja, ik ontvang gepersonaliseerd beweegadvies
☐ Anders, namelijk…

[PAGE BREAK]

20. Technology use (hardware)

NB. Not used as variable in the study reported in the manuscript

Welke apparaten heeft u? (Meerdere antwoorden mogelijk)

☐ Een tablet
☐ Een smartphone
☐ Een smartwatch
☐ Een stappenteller
☐ Anders, namelijk…

[PAGE BREAK]

21. Technology use (software)

NB. Not used as variable in the study reported in the manuscript

Maakt u gebruik van apps op uw tablet, smartphone of mobiele telefoon?

☐ Ja
☐ Nee

[PAGE BREAK]

Onderdeel 3. Demografie

Ten slotte zijn we nog geïnteresseerd in een aantal demografische gegevens.

22.Gender

Ik ben een:
☐ Man
☐ Vrouw

[PAGE BREAK]

23.Age

Wat is uw leeftijd?
.....

[PAGE BREAK]

24. Number of people in household

Uit hoeveel personen bestaat uw huishouden?
... personen, waarvan ... kinderen onder de 18 jaar.

[PAGE BREAK]

25.Education

Wat is het hoogste opleidingsniveau dat u hebt voltooid?
LO (lagere school, LAVO, VGLO)
LBO (LBO, LTS, ITO, LEAO, Huishoudschool, LLO)
MAO (MAVO, IVO, MULO, ULO, 3jr HBS, 3jr VWO, 3jr VHMO)
MBO (MTS, UTS, MEAO)
HAO (HAVO, VWO, Atheneum, Gymnasium, NMS, HBS, Lyceum)
HBO (HTS, HEAO, Wetensch. kand., Univers. onderwijs kand.)
WO (Universitair onderwijs, Doctoraalopleiding, TH)

Note: The categorisation of the Dutch education system is used. The colours indicate low, medium and high education level.

[PAGE BREAK]

26.Net monthly income

Wat is het netto maandinkomen van uw gezin?
Minder dan € 1.000
€ 1.000 tot € 1.500
€ 1.500 tot € 2.000
€ 2.000 tot € 3.000
€ 3.000 tot € 5.000
€ 5.000 tot € 7.500
Meer dan € 7.500
Weet ik niet / Zeg ik liever niet

Note: Dutch income categories are used. The colours indicate low, medium and high net monthly income level.

[PAGE BREAK]

27.Work status

Wat is uw huidige werksituatie? ITEMS RANDOM

☐ Zelfstandige (en ik heb werknemers in dienst)
☐ Zelfstandige (ik werk alleen)
☐ Voltijds werknemer
☐ Parttime werknemer
☐ Tijdelijke werknemer/seizoenswerknemer
☐ Voltijds huisvrouw/huisman
☐ Voltijds student
☐ Werkeloos
☐ Gepensioneerd
☐ Anders

[PAGE BREAK]

28.Final mark

Kunt u tot slot een rapportcijfer geven voor de vragenlijst (Geef een cijfer van 1-10):

Moeilijkheid (1=heel moeilijk, 10 = heel makkelijk) …………..
Interessant (1=heel saai, 10 = heel interessant) …………..
Lengte (1=veel te lang, 10=helemaal niet te lang) …………..

Hartelijk dank voor uw medewerking!
