# Supplementary material for: Consumer acceptance of personalised nutrition: The role of ambivalent feelings and eating context
Source: PLoS One. 2020 Apr 13;15(4):e0231342. doi: 10.1371/journal.pone.0231342 (PMC7153894; doi:10.1371/journal.pone.0231342)
Supplement: S2 Data — Translated questionnaire (in English). (PDF) [file pone.0231342.s002.pdf]

# PERSONALISED NUTRITION – (TRANSLATED) ENGLISH QUESTIONNAIRE

Welcome to the study!

This study is being carried out on behalf of Wageningen University & Research as part of a long-term research consortium. **In this study, we are interested in your choices and views on personalised nutrition advice**. Participation is voluntary and your responses will remain anonymous. Only the partners in the research consortium will be able to access the (anonymised) data.

### Instruction

- All possible answers are shown with the questions. Always tick the box for the answer that most applies to you.
- You are only supposed to tick one box. If several answers can be given, this is indicated with the question.
- Diverse keren wordt gevraagd aan te geven in hoeverre u het (on)eens bent met een aantal uitspraken. Als de stelling helemaal niet kenmerkend is voor u, vink dan een “1” aan; als de stelling helemaal wel kenmerkend is voor u, vink dan een “7” aan. En gebruik de cijfers in het midden als uw antwoord tussen deze twee extremen valt.
- You will be asked several times to indicate to what extent you (dis)agree with a number of statements. If you (totally) disagree with the statement, tick a "1"; if you (totally) agree with the statement, tick a "7". And use the numbers in the middle if your answer falls between these two extremes.
- Filling out this questionnaire takes approximately 20 minutes.

**NB: You may find some of the questions difficult to answer. If that is the case, please try to respond nonetheless. There are no right or wrong answers; we are interested in your (first) impression.**

**Please proceed if you agree with participation.**

[PAGE BREAK]

# Part 1. Personalised nutrition advice

This questionnaire is about personalised nutrition advice. Personalised nutrition advice is advice that is completely tailored to you as a person. Advice can be personalised in different ways:

- personal preferences (e.g., what food you like and don't like),
- self-formulated goals/ wishes (e.g., lowering cholesterol; enhancing fruit consumption),
- eating habits,
- health status (e.g., overweight, blood pressure and cholesterol),
- or even DNA profile.

Information technology developments ride on this personalisation trend with 'health trackers', like smart watches, pedometers and smartphone apps, which help you to get insight in your own behaviour or health.

An example:

- Generic nutrition advice: general nutrition guidelines prescribe you to eat whole grain products at a daily basis.
- Personalised nutrition advice: based on your personal profile and preferences we advise you to eat yoghurt with oatmeal instead of whole grain bread.

With the following questionnaire we want to know more about your opinion on personalised nutrition advice.

[PAGE BREAK]

## *1. Understanding definition question*[1]

| ITEMS RANDOM | Not at all 1 | 2 | 3 | Neutral 4 | 5 | 6 | Totally 7 |
|---|---|---|---|---|---|---|---|
| Do you have a clear picture what personalised nutrition advice means based on the provided definition? | ☐ | ☐ | ☐ | ☐ | ☐ | ☐ | ☐ |

[PAGE BREAK]

## *2. Intention to make use of personalised nutrition advice*

Please indicate to what extent you agree/ disagree with the following statements:

| ITEMS RANDOM | Strongly disagree 1 | 2 | 3 | Neutral 4 | 5 | 6 | Strongly agree 7 |
|---|---|---|---|---|---|---|---|
| 1. I intend to use personalised nutrition advice. | ☐ | ☐ | ☐ | ☐ | ☐ | ☐ | ☐ |
| 2. I would consider using personalised nutrition advice. | ☐ | ☐ | ☐ | ☐ | ☐ | ☐ | ☐ |
| 3. I am definitely going to use personalised nutrition advice. | ☐ | ☐ | ☐ | ☐ | ☐ | ☐ | ☐ |

[PAGE BREAK]

## *3. Ambivalent Feelings*

When I would use personalised nutrition advice…

ITEMS RANDOM

| | 1 | 2 | 3 | 4 | 5 | 6 | 7 | |
|---|---|---|---|---|---|---|---|---|
| I feel no conflict at all | ☐ | ☐ | ☐ | ☐ | ☐ | ☐ | ☐ | I feel maximum conflict |
| I feel no uneasiness at all | ☐ | ☐ | ☐ | ☐ | ☐ | ☐ | ☐ | I feel maximum uneasiness |
| I have no mixed feelings | ☐ | ☐ | ☐ | ☐ | ☐ | ☐ | ☐ | I have strong mixed feelings |

[1] These headings are added for the researchers (so that they know what construct/ concept the specific question belongs to), but were not shown to the participants.

[PAGE BREAK]

## 4. *Benefits & risks personalised nutrition*

Items 1-3: Personalisation Benefit
Items 4-6: Service Effectiveness > *NB. Not used as variable in the study reported in the manuscript*
Items 7-9: Privacy Risk

| Compared to general nutrition advice, personalised nutrition advice offers me advice that...<br>ITEMS RANDOM | Strongly disagree<br>1 | 2 | 3 | Neutral<br>4 | 5 | 6 | Strongly agree<br>7 |
|---|---|---|---|---|---|---|---|
| 1. ...Is more accurately tailored to my health needs | ☐ | ☐ | ☐ | ☐ | ☐ | ☐ | ☐ |
| 2. ...Is more relevant for my health | ☐ | ☐ | ☐ | ☐ | ☐ | ☐ | ☐ |
| 3. ...Is more beneficial for my health | ☐ | ☐ | ☐ | ☐ | ☐ | ☐ | ☐ |
| 4. ...Enables me to develop a healthier lifestyle | ☐ | ☐ | ☐ | ☐ | ☐ | ☐ | ☐ |
| 5. ...Helps me to have a healthier lifestyle | ☐ | ☐ | ☐ | ☐ | ☐ | ☐ | ☐ |
| 6. ...Makes me feel in control of developing a healthier lifestyle | ☐ | ☐ | ☐ | ☐ | ☐ | ☐ | ☐ |
| 7. ...Involves many privacy-related risks | ☐ | ☐ | ☐ | ☐ | ☐ | ☐ | ☐ |
| 8. ...Is a threat to my privacy | ☐ | ☐ | ☐ | ☐ | ☐ | ☐ | ☐ |
| 9. ...Creates a high risk for the loss of my privacy | ☐ | ☐ | ☐ | ☐ | ☐ | ☐ | ☐ |

[PAGE BREAK]

## 5. *Perceived benefits*

*NB. Not used as variable in the study reported in the manuscript*

| What of the following benefits would increase your uptake of personalised nutrition?<br>ITEMS RANDOM | Strongly disagree<br>1 | 2 | 3 | Neutral<br>4 | 5 | 6 | Strongly agree<br>7 |
|---|---|---|---|---|---|---|---|
| 1. Knowing what foods are best | ☐ | ☐ | ☐ | ☐ | ☐ | ☐ | ☐ |
| 2. Losing weight | ☐ | ☐ | ☐ | ☐ | ☐ | ☐ | ☐ |
| 3. Gaining weight | ☐ | ☐ | ☐ | ☐ | ☐ | ☐ | ☐ |
| 4. Fitness | ☐ | ☐ | ☐ | ☐ | ☐ | ☐ | ☐ |
| 5. Improving family's health | ☐ | ☐ | ☐ | ☐ | ☐ | ☐ | ☐ |
| 6. Improving health | ☐ | ☐ | ☐ | ☐ | ☐ | ☐ | ☐ |
| 7. Improving quality of life | ☐ | ☐ | ☐ | ☐ | ☐ | ☐ | ☐ |
| 8. Improving sports performance | ☐ | ☐ | ☐ | ☐ | ☐ | ☐ | ☐ |
| 9. Improving skin condition | ☐ | ☐ | ☐ | ☐ | ☐ | ☐ | ☐ |
| 10. Preventing a future illness | ☐ | ☐ | ☐ | ☐ | ☐ | ☐ | ☐ |
| 11. Preventing expression of hereditary illness | ☐ | ☐ | ☐ | ☐ | ☐ | ☐ | ☐ |

[PAGE BREAK]

## 6. *Barriers personalised nutrition (1) – data protection*

*NB. Not used as variable in the study reported in the manuscript*

| Please indicate to what extent you agree/ disagree with the following statements:<br>ITEMS RANDOM | Strongly disagree<br>1 | 2 | 3 | Neutral<br>4 | 5 | 6 | Strongly agree<br>7 |
|---|---|---|---|---|---|---|---|
| 1. I worry that a personalised diet plan is not effective. | ☐ | ☐ | ☐ | ☐ | ☐ | ☐ | ☐ |
| 2. I worry about how my personal data might be used by authorities. | ☐ | ☐ | ☐ | ☐ | ☐ | ☐ | ☐ |
| 3. I worry that my personal data may not be treated confidentially. | ☐ | ☐ | ☐ | ☐ | ☐ | ☐ | ☐ |
| 4. I worry about how my personal data and test results might be stored. | ☐ | ☐ | ☐ | ☐ | ☐ | ☐ | ☐ |
| 5. I worry about how my personal data might be used by personalised nutrition providers. | ☐ | ☐ | ☐ | ☐ | ☐ | ☐ | ☐ |
| 6. I worry about how my personal data might be used by advertisers. | ☐ | ☐ | ☐ | ☐ | ☐ | ☐ | ☐ |
| 7. I worry about how my personal data might be used by insurance companies. | ☐ | ☐ | ☐ | ☐ | ☐ | ☐ | ☐ |
| 8. I worry that my personal data might be accessed by hackers. | ☐ | ☐ | ☐ | ☐ | ☐ | ☐ | ☐ |

[PAGE BREAK]

## 7. Barriers personalised nutrition (2)

Items 1-5: Eating context barrier
Items 6-7: Liking of food barrier > *NB. Not used as variable in the study reported in the manuscript*
Items 8-10: Social context barrier > *NB. Not used as variable in the study reported in the manuscript*

| What would prevent you from using personalised nutrition advice? ITEMS RANDOM | Strongly disagree 1 | 2 | 3 | Neutral 4 | 5 | 6 | Strongly agree 7 |
|---|---|---|---|---|---|---|---|
| 1. Providing different foods for family members. | ☐ | ☐ | ☐ | ☐ | ☐ | ☐ | ☐ |
| 2. Difficulties in maintaining healthy eating habits when eating out in restaurants. | ☐ | ☐ | ☐ | ☐ | ☐ | ☐ | ☐ |
| 3. Difficulties in maintaining healthy eating habits when eating at other people's houses. | ☐ | ☐ | ☐ | ☐ | ☐ | ☐ | ☐ |
| 4. Difficulties in maintaining diet when travelling. | ☐ | ☐ | ☐ | ☐ | ☐ | ☐ | ☐ |
| 5. Difficulties maintaining diet when at work. | ☐ | ☐ | ☐ | ☐ | ☐ | ☐ | ☐ |
| 6. Being told to eat foods you don't like. | ☐ | ☐ | ☐ | ☐ | ☐ | ☐ | ☐ |
| 7. Not being recommended to eat foods you like. | ☐ | ☐ | ☐ | ☐ | ☐ | ☐ | ☐ |
| 8. My family rejecting the adoption of personalised nutrition. | ☐ | ☐ | ☐ | ☐ | ☐ | ☐ | ☐ |
| 9. My friends rejecting the adoption of personalised nutrition. | ☐ | ☐ | ☐ | ☐ | ☐ | ☐ | ☐ |
| 10. Society rejecting the adoption of personalised nutrition. | ☐ | ☐ | ☐ | ☐ | ☐ | ☐ | ☐ |

[PAGE BREAK]

## 8. Trust in agencies to provide information on personalised nutrition

*NB. Not used as variable in the study reported in the manuscript*

| To what extent do you trust the following providers of personalised nutrition advice? ITEMS RANDOM | Totally not 1 | 2 | 3 | Neutral 4 | 5 | 6 | Totally 7 |
|---|---|---|---|---|---|---|---|
| 1. Supermarkets | ☐ | ☐ | ☐ | ☐ | ☐ | ☐ | ☐ |
| 2. Food manufacturers | ☐ | ☐ | ☐ | ☐ | ☐ | ☐ | ☐ |
| 3. Commercial providers of personalised nutrition advice | ☐ | ☐ | ☐ | ☐ | ☐ | ☐ | ☐ |
| 4. Commercial providers of technology | ☐ | ☐ | ☐ | ☐ | ☐ | ☐ | ☐ |
| 5. Popular diets (books) | ☐ | ☐ | ☐ | ☐ | ☐ | ☐ | ☐ |
| 6. Universities | ☐ | ☐ | ☐ | ☐ | ☐ | ☐ | ☐ |
| 7. Dieticians | ☐ | ☐ | ☐ | ☐ | ☐ | ☐ | ☐ |
| 8. Personal trainers / gym | ☐ | ☐ | ☐ | ☐ | ☐ | ☐ | ☐ |
| 9. General practitioner | ☐ | ☐ | ☐ | ☐ | ☐ | ☐ | ☐ |
| 10. Hospital | ☐ | ☐ | ☐ | ☐ | ☐ | ☐ | ☐ |
| 11. Municipal health services | ☐ | ☐ | ☐ | ☐ | ☐ | ☐ | ☐ |
| 12. Dutch Ministry of Health | ☐ | ☐ | ☐ | ☐ | ☐ | ☐ | ☐ |
| 13. National nutrition centra | ☐ | ☐ | ☐ | ☐ | ☐ | ☐ | ☐ |
| 14. Rijksinstituut voor Volksgezondheid en Milieu (RIVM) | ☐ | ☐ | ☐ | ☐ | ☐ | ☐ | ☐ |
| 15. Friends | ☐ | ☐ | ☐ | ☐ | ☐ | ☐ | ☐ |
| 16. Family/ relatives | ☐ | ☐ | ☐ | ☐ | ☐ | ☐ | ☐ |
| 17. My employer | ☐ | ☐ | ☐ | ☐ | ☐ | ☐ | ☐ |

[PAGE BREAK]

## 9. Risk-Benefit Calculus

| ITEMS RANDOM | Greater risks 1 | 2 | 3 | Neutral 4 | 5 | 6 | Greater benefits 7 |
|---|---|---|---|---|---|---|---|
| All things considered, do you think using personalised nutrition advice will offer greater benefits than risks, or greater risks than benefits? | ☐ | ☐ | ☐ | ☐ | ☐ | ☐ | ☐ |

[PAGE BREAK]

# Part 2. Background characteristics

## 9. *Intention to eat healthily/ stay eating healthily*

*NB. Not used as variable in the study reported in the manuscript*

| Please indicate to what extent you agree/ disagree with the following statements:<br><br>ITEMS RANDOM | Strongly disagree<br>1 | 2 | 3 | Neutral<br>4 | 5 | 6 | Strongly agree<br>7 |
|---|---|---|---|---|---|---|---|
| 1. I intend to eat healthily/ stay eating healthily. | ☐ | ☐ | ☐ | ☐ | ☐ | ☐ | ☐ |
| 2. I would consider eating healthily/to stay eating healthily. | ☐ | ☐ | ☐ | ☐ | ☐ | ☐ | ☐ |
| 3. I am definitely going to eat healthily/ stay eating healthily. | ☐ | ☐ | ☐ | ☐ | ☐ | ☐ | ☐ |

[PAGE BREAK]

## 10. *Subjective knowledge*

*NB. Not used as variable in the study reported in the manuscript*

| Please indicate to what extent the following statements apply to you<br><br>ITEMS RANDOM | Totally not<br>1 | 2 | 3 | Neutral<br>4 | 5 | 6 | Totally<br>7 |
|---|---|---|---|---|---|---|---|
| 1. I know a lot about healthy food. | ☐ | ☐ | ☐ | ☐ | ☐ | ☐ | ☐ |
| 2. In my circle of friends, I am one of the 'experts' in the field of healthy food. | ☐ | ☐ | ☐ | ☐ | ☐ | ☐ | ☐ |

[PAGE BREAK]

## 12. *Self-efficacy*

*NB. Not used as variable in the study reported in the manuscript*

| | Very difficult<br>1 | 2 | 3 | Neutral<br>4 | 5 | 6 | Very easy<br>7 |
|---|---|---|---|---|---|---|---|
| How easy would it be for you to start eating healthily if you want to? | ☐ | ☐ | ☐ | ☐ | ☐ | ☐ | ☐ |

[PAGE BREAK]

## 13. *Lifestyle*

*NB. Not used as variable in the study reported in the manuscript*

Do you follow a certain dietary guideline/ rule? (More answers possible)

☐ No
☐ Yes, I am flexitarian (sometimes/ not always meat)
☐ Yes, I am vegetarian (no meat)
☐ Yes, I am vegan (no animal-based products)
☐ Yes, because of my religion
☐ Other……………………

[PAGE BREAK]

## *14. Food-related health problem*

*NB. Not used as variable in the study reported in the manuscript*

Do you have a food-related health problem? (More answers possible)
☐ No
☐ Yes, diabetes type II
☐ Yes, high blood pressure
☐ Yes, overweight/ obesity
☐ Yes, liver problems
☐ Yes, constipation
☐ Yes, high cholesterol
☐ Other...

[PAGE BREAK]

## *15. Diet*

*NB. Not used as variable in the study reported in the manuscript*

Did you follow a diet last month? (More answers possible)
☐ No
☐ Yes, an energy-restricting diet (slimming diet)
☐ Yes, a sodium-restricted diet (low-salt diet)
☐ Yes, a fat-restricted diet
☐ Yes, a fiber-rich diet
☐ Yes, a diet for diabetes mellitus
☐ Yes, a diet for high cholesterol
☐ Other.......................

[PAGE BREAK]

## *16. Subjective health*

*NB. Not used as variable in the study reported in the manuscript*

| Please indicate to what extent the following statements apply to you: | Very unhealthy 1 | 2 | 3 | Neutral 4 | 5 | 6 | Very healthy 7 |
|---|---|---|---|---|---|---|---|
| ITEMS RANDOM | | | | | | | |
| 1. How healthy do you think you are? | ☐ | ☐ | ☐ | ☐ | ☐ | ☐ | ☐ |
| 2. How healthy do you think your diet is in general (think of the last year)? | ☐ | ☐ | ☐ | ☐ | ☐ | ☐ | ☐ |

[PAGE BREAK]

## *17.BMI*

*NB. Not used as variable in the study reported in the manuscript*

What is your height in centimeters?
...
What is your weight in kilograms?
...

[PAGE BREAK]

## 18. Shopping

*NB. Not used as variable in the study reported in the manuscript*

| The following questions are about groceries. ITEMS RANDOM | Never 1 | Rarely 2 | Occasionally 3 | Regularly 4 | Always 5 |
|---|---|---|---|---|---|
| 1. How often do you do the shopping? | ☐ | ☐ | ☐ | ☐ | ☐ |
| 2. How often do you shop online? | ☐ | ☐ | ☐ | ☐ | ☐ |

[PAGE BREAK]

## 19. Using personalised nutrition services

*NB. Used as a selection question in the study: participants who indicated that they already use some form of personalised nutrition were omitted from the analyses, to avoid a bias in the results by people that already use personalised nutrition advice who may see benefits and risks differently based on their experiences.*

Do you make use of personalised services?

☐ No
☐ Yes, I receive personalised nutrition/ food advice
☐ Yes, I receive personalised exercise/ fitness advice
☐ Yes, other…

[PAGE BREAK]

## 20. Technology use (hardware)

*NB. Not used as variable in the study reported in the manuscript*

Which device(s) do you use? (More answers possible)

☐ A tablet
☐ A smartphone
☐ A smartwatch
☐ A pedometer
☐ Otherwise, namely…

[PAGE BREAK]

## 21. Technology use (software)

*NB. Not used as variable in the study reported in the manuscript*

Do you make use of apps on your tablet, smartphone or mobile phone?

☐ Yes
☐ No

[PAGE BREAK]

# Part 3. Demographics

## 22.Gender

I am:
☐ Male
☐ Female

[PAGE BREAK]

## 23.Age

What is your age? (in years)
.....

[PAGE BREAK]

## 24. Number of people in household

**How many people does your household consist of?**
... persons, of which ... children under 18 years of age.

[PAGE BREAK]

## 25.Education

| What is the highest level of education you have completed? |
|---|
| LO (Primary school) |
| LBO (Lower vocational education) |
| Lower secondary education and pre-vocational secondary education (VMBO, MAVO, 3jr VWO, 3jr HAVO) |
| MBO (Vocational Education) |
| Higher secondary education (HAVO, VWO, Atheneum, Gymnasium) |
| HBO (University of Professional Education) |
| WO (University, polytechnic) |
| Postgraduate degree |

*Note: The categorisation of the Dutch education system is used. The colours indicate low, medium and high education level.*

*See below for comparable British education levels:*

| What is the highest level of education you have completed? |
|---|
| No school |
| Primary |
| Some secondary school |
| 'O' levels/CSE/GCSE |
| 'A' levels/HND/Highers |
| Technical/trade studies/GNVQ (incomplete) |
| Technical/trade studies/GNVQ (complete) |
| Some university/polytechnic (incomplete) |
| University/polytechnic graduate |
| Some postgraduate studies |
| Postgraduate degree |

[PAGE BREAK]

## 26.Net monthly income

**What is your household's net monthly income?**

Less than € 1.000

€ 1.000 to € 1.500

€ 1.500 to € 2.000

€ 2.000 to € 3.000

€ 3.000 to € 5.000

€ 5.000 to € 7.500

More than € 7.500

I don't know / I'd rather not say

*Note: Dutch income categories are used. The colours indicate low, medium and high net monthly income level.*

[PAGE BREAK]

## 27.Work status

What is your current work status? ITEMS RANDOM

☐ Self-employed (with employees)
☐ Self-employed (without employees)
☐ Fulltime employee
☐ Parttime employee
☐ Temporary/ seasonal work
☐ Fulltime housewife/-husband
☐ Fulltime student
☐ Unemployed
☐ Retired
☐ Other

[PAGE BREAK]

## 28.Final mark

Finally, can you give a mark for the questionnaire (Please give a mark from 1-10)

Difficulty (1= very difficult, 10 = very easy) …………..
Attractiveness (1= very boring, 10 = very interesting) …………..
Length (1= far too long, 10= not too long at all) …………..

**Thank you for your participation!**
