# Supplementary material for: Consumer acceptance of personalised nutrition: The role of ambivalent feelings and eating context
Source: PLoS One. 2020 Apr 13;15(4):e0231342. doi: 10.1371/journal.pone.0231342 (PMC7153894; doi:10.1371/journal.pone.0231342)
Supplement: Attachment — Submitted filename: PLOS ONE review of Consumer Acceptance of PNS Nov 2019.docx [file pone.0231342.s004.docx]

PLOS ONE review of:

CONSUMER ACCEPTANCE OF PERSONALISED NUTRITION: THE ROLE OF AMBIVALENT FEELINGS AND EATING CONTEXT

L = Line, p = page

Summary of the Research: Overall, this concept of ‘Personalised Nutrition Services’ sounds interesting. It may have potential to influence some individuals who are interested in these types of apps or technology. However, the biggest issue I have with this article is the questionnaire. I do not understand exactly how the questions are phrased. I feel it is important that the authors include the original questionnaire they used for the survey if the reader is to follow the authors’ explanations and results. Specifically because the authors discuss (p 10) the 7-point Likert scale of the questionnaire ranging from 1: strongly disagree to 7: strongly agree. But then in Table 2, for example, scale number 3 ‘Ambivalent feelings’ has three items: ‘I feel no conflict at all/I feel maximum conflict’. From the way the authors present their work, I cannot determine how a participant’s response to this question is to be interpreted. In other words, does the participant ‘strongly agree or disagree’ with ‘feeling no conflict or maximum conflict’ what does or how is the respondent’s answer interpreted? Another way of stating my question is: Is the Likert scale response 1: ‘strongly disagree’ to 7: ‘strongly agree’, or is it, 1: ‘I feel no conflict at all’ to 7: I feel maximum conflict’? Including the original questionnaire may help to clarify the answer to this question.

In addition, p 10 should be written more succinctly. The authors need only to state once that all scales are based on a seven-point Likert scale response, and then describe each question or scale. As an example lines 175-176 would be clearer if the authors wrote something more similar along the lines of: “Privacy Risk was measured using 3-items which explored issues around privacy and privacy-related risks, and was based on Berezowska” etc. In trying to explain this questionnaire, it might serve the authors better if they include the questionnaire in a box or table and then discuss it that way.

Moreover, all responses, 1 through 7, should be listed as to what each response equates to, because I do not know what a response of 3 or 4 equates to (ex. L 217, p 13) The authors need to be clear about what the ‘mean’ suggests, in terms of understanding how to interpret these scales accurately.

The authors need to be clearer on how the results are interpreted. In other words, it is not enough to report ‘our results show’. Results need to be explained.

Below, is feedback on some issues I have about the article. Please take it on board as constructive criticism and helpful feedback.

ABSTRACT:

L 12: The first line of the abstract the author uses the word ‘can’ contribute to the prevention of non-communicable diseases… can is a strong word, is there proof of this? I suggest using the word ‘may’ unless you have strong scientific proof where you can back it up with references.

L 25: …the current study ‘shows’… this current study can only ‘suggest’, as it takes proof to ‘show’ that there is a connection between one thing and another thing.

L 28: instead of using the word “someone’s”, may I suggest that you use “individual’s”, and this would be the case throughout the rest of the article when you use ‘someone’s, as this expression is not scientific.

INTRODUCTION:

L 34 (p. 3), instead of ‘needs by so-called’ it is preferable to express it as ‘needs by what have been termed ‘personalised nutrition services’ …and using the brackets to demarcate the term. Also, you expressed that it has ‘great’ potential, is this exactly what your reference Celis-Morales et al. 2015 expressed? Or is that your opinion? I would be careful about using emotive expressions because they are like promises you cannot possibly keep.

L 37 (p. 3) ‘obesity have not been very successful’… does the WHO 2011 reference actually report this? I suggest finding a more scientific way of reporting this, such as: ‘One-size-fits-all attempts to turn the tide of obesity have not been successful at reducing the prevalence of obesity’ (or something along those lines). Again, it is just removing the strong emotive words, or opinionated or watered-down words.

L 39 (p 3) ‘personalized’ is correct but you need to be consistent in all your spellings. In other words, you should spell it as ‘personalised’ since that is generally how you spell it throughout your paper.

L 41 (p 3) ‘Personalisation will make’… it is better to express it as ‘Personalisation has the potential to make the provided’…

L 45 (p 3) instead of using the word ‘like’ it is better to say ‘such as’

L 47 – L51 (p 3) is a run-on sentence, break it up into at least 2 sentences. Also, do you have a reference for the sentences of Lines 47 through 53? Otherwise, it sounds like it is your opinion that consumer interest is growing. How do you know consumer interest is growing?

L 54 (p 3) do you have a reference about ‘personalised nutrition services’ as having ‘great potential’ and ‘consumer reluctance’, both these need to be supported by a reference.

L 55 (p 3) ‘crucial’ sounds a bit too strong, my suggestion is ‘it would be advantageous to understand’… Also, what are the references for consumers’ lack of knowledge about these devices or apps? Doesn’t that play a role? Consumer reluctance implies that ‘all consumers’ are aware of these devices or apps but are reluctant to use them.

L 61 & 64 & 78, 109 etc (p 4) same comment as expressed earlier in L 25, it is hard to prove that a study ‘shows’ anything, at best, a study suggests or may indicate… this comment is the same for other lines where you use the word ‘show’.

L 62 (p 4) ‘and the less the perceived risks’… there are possibly too many ‘thes’ in this sentence, I’d remove the second ‘the’, or technically the fourth ‘the’ in that sentence.

L 63 (p 4) I think you mean ‘the greater is the consumers’ intention to adopt’ (as this reads better or more clearly)

L 64-67 I’m not certain I understand this sentence. Do you mean that individuals are not willing to enrol in personalised nutrition services because they feel they are putting their privacy at risk? If this is the case, I would take these words ‘and putting at risk their privacy’ out of the parentheses and re-word the sentence. I suggest something along the line of: ‘Consumers feel that disclosing their privacy information is too big of a risk compared to the benefits that personalised nutrition services can offer’.

L 68 (p 4) instead of saying ‘they call’ say ‘they have termed it’, or ‘which has been termed’. Also L67-71 is a run-on sentence. Personally, I would place a period after the parentheses and start a new sentence with ‘This Privacy Calculus is…’

L 71-75 is a run-on sentence… I do not understand this sentence, I think it’s because you’re trying to say too much in one sentence. Break up in individual sentences what it is you’re trying to convey.

L 95 (p 5) ‘is perceived to have both benefits’ do you mean factors? Is having food neophobia a benefit, or food neophilia a benefit. (I know I am knit-picking here, but your choice of words has to be concise because words convey an image and meaning, and I’m not sure that either food neophobia or neophilia can be seen as ‘benefits’, I see them as factors that have to be considered when making these personalised nutrition apps or devices, in order to ‘personalise’ nutrition suggestions.

L 99 & 115 (p 5 & 6) should be written as: consumers’ adoption

L 110-111 (p 6) ‘in adhering to a personalised advice’, remove ‘a’; ‘eating at other people’s houses’ instead perhaps say it as ‘eating in friends’ or acquaintances’ homes. Also, remove the s on advices. Advice can suggest both the singular and plural. Instead of ‘ideally take the preferences’, should instead perhaps be written as: ‘would benefit from considering the eating preferences of others in the eating context over the preference of the individual’.

My own comment on the above sentence is that it renders ‘personalised nutrition services’ as no longer being ‘personalised’. Perhaps there should be an app or device that is called something different in order to service these needs, such as ‘group or context nutrition services’…

L 116-117 (p 6) sentence might be better if written as: ‘to build on previous research, (which has investigated personalised nutrition advice), by exploring’…

L 118-119 (p 6) this sentence would be crisper if you added ‘factors help to further explain’… ‘Additionally, our work sheds light into how these factors may relate’…

L 121 (p 6) instead of saying ‘article’, perhaps work or research is a better expression, also findings ‘may potentially’ be used to design personalised…

METHODS section (p 6 & 7) needs to be written more scientifically. Also, it would be helpful if the authors state exactly what a response of 4 equates to (L 142).

L 144 (p 7) you state that 797 respondents comprise your sample, but your abstract states that your data was from 996 (L 17, p 2) respondents?

L 146 (p 7) what is ‘low, moderate and high’ education?

The box on page 9 needs a legend. Additionally, the example in the box for ‘Personalised nutrition advice’ seems very generic as well. Perhaps not as generic as prescribing eating whole grain products. But the ‘Personalised nutrition advice to eat yoghurt with oatmeal instead’ could be perceived by respondents as too generic and not personal to them. In my opinion, personalised nutrition advice should be specifically making suggestions about how many grams of fibre and/or calcium or vitamin D and B vitamins, magnesium, etc., a day an individual is getting (based on the example here of why a person should choose oatmeal and yoghurt since it contains all these micro-nutrients). And again, it is just my opinion, based on this example, that my first impression is that I see ‘personalised nutrition advice’ as a gimmick, and my gut tells me not to trust it. However, from your questionnaire you have no way of knowing if any of your respondents may have felt this way as well. And, as a result, I think you need to be very careful how you interpret your results. Furthermore, you should not use expressions such as, ‘our results show or imply’ (as in L220, p 13) because again, your results cannot show, they can only suggest what you think the interpretation is...

DATA ANALYSIS:

Page 12 I do not feel I have enough experience in this respect to make any comments, the only question or comment I can make with respect to this section is: Did you carry out a normalcy distribution test on your data? I do not think you can assume that your data is normally distributed, as is suggested by reporting the mean and standard deviation. If your data is not normally distributed, how can you know that mean and standard deviation is the best way to present your data? As median and interquartile range may be a better way to present it. It might change the results of your findings. Also, looking at ‘mode’ can also be a different way to investigate your data. By looking at just the average (or the mean), the average has a way of watering down or equalising what you might be finding. You might be missing important details if you are looking at just one data point (as in, looking only at the mean).

RESULTS:

L 216 (p 13), you write that ‘respondents provided the highest average scores’. Respondents did not ‘provide’, it is more accurate to report that ‘respondents had the highest mean score’…

L 217 (p 13) what does a mean score of 5.16 suggest? How is a score of 4.88 interpreted exactly?

L 220 (p 13) again, your results cannot imply anything, but only suggest.

STRUCTURAL MODELS ESTIMATION:

L 234 -276 (pp 14-16) It would be helpful if you write exactly what it means when items are positively or negatively associated. Clearly explain what these correlations suggest. In other words, what does it mean when you write that ‘Personalisation Benefit was negatively related to Ambivalent Feelings? Does it suggest that as an individual’s ambivalent feelings increase their personalisation benefits decrease? And if this is the case, clearly state it. If the results are not clearly stated, it is my opinion that there is too much room for ambiguity in your results and you leave it to the reader to decide what the findings mean. Personally, I find myself getting lost in what the findings mean, and I don’t know that I necessarily trust the authors’ opinion on their results mainly because the structure and phrase-ology of the questionnaire is not clear. Listing each item’s mean and SD scores and explaining exactly how each score is interpreted, may help me as a reader to understand better how the questionnaire items can be understood. I find that I am confused about aspects related to, as an example, privacy risk and benefit: Do the respondents think that ‘personalised nutrition advice’ is a risk, or do they think it benefits them.

L 278 (p 16) the figure is missing. However, I found it on the back of the last page, after the references. Not sure why it wasn’t where the figure legend is written.

DISCUSSION: This section also needs to be written in better scientific language

L 291 (p 16): ‘First, the current study confirms previous research’, using the word ‘confirms’ is not accurate. Your study may be in agreement with previous research, but it does not confirm it. Specifically, how did previous research take their measures? What were their methods? Did they use the same questionnaire as you used to take your measures? Also, as I previously mentioned about your findings and when you discuss them, you have to be careful how you interpret what you have found. Because there may be other factors at play that you (or other researchers) have not considered or thought about, such as if individuals ‘think’ or ‘feel’ that personalised nutrition is a gimmick, or perhaps they feel it is too generic, or it just does not pertain to them. They may feel that personalised nutrition does not offer them anything new. Additionally, why did you not measure or consider those who know nothing about personalised nutrition? Wouldn’t it be advantageous to understand why this group have no knowledge of personalised nutrition? Isn’t this lack of knowledge another barrier for individuals accessing or using personalised nutrition apps or devices? And, on a similar vein, what about those who have used personalised nutrition advice? What were their thoughts and feelings regarding it? I think you have too conveniently brushed these participants aside. Although these two groups comprise fewer participants, it would be interesting to compare (and comment) on these outcomes as well.

L 300-304 (p 17) I do not understand this sentence probably because I do not understand what you mean by ‘affective laden construct’. Can you describe what you mean by using that term? Also, your study can also suggest, not show. Also, you use the word ‘fact’ on occasion, and ‘fact’ is something you have to prove. Facts are hard to prove, so my suggestion is that you express it in terms of in Line 304 ‘These findings suggest that conflicting’…

L 307 (p 17): ‘The fact that ambivalent feelings negatively predict usage intention indicates that ambivalence is experienced as a negative, aversive state’. I do not know that I can agree with this statement, particularly because I do not understand exactly how the questions in your questionnaire have been termed or how exactly they have been written. It would seem to me, that depending on how the questions were written, you should be able to untangle the exact feelings of each respondent. In other words, you should be able to separate out the individuals who have ‘positive’ feelings towards ‘personalised nutrition advice’ from individuals who have ‘negative’ feelings. It would seem to me that your data analysis would be better analysed if you allocate those participants with high scores from those with low scores, and then see what the correlation results are.

L 328 (p 18): ‘important predictor of someone’s intention to use personalised nutrition advice. Besides an’ … instead should be written as: ‘important predictor of an individual’s intention to use personalised nutrition advice. Other than an’… and ‘few’ studies, as opposed to what you have written ‘not many’ studies.

L 340 (p 18) & L 363 (p 19) spelling of individualized or individualised, and personalized or personalise, be consistent in your spellings.

LIMITATIONS AND FUTURE RESEARCH: Needs to be written in better scientific language. As an example L350-351 (p 19): ‘A number of limitations exist in this study, and we include suggestions for future research. Firstly, self-reported measures were used to identify’…

L 363-368 all need improving… additionally, I do not understand what you are saying when you write: L 363-364 (p 19): ‘This general description of personalised nutrition could have led to that respondents may have reacted to different “products”.

I suggest writing L364-368: ‘As an example, it is possible that perceived risks may be more important for personalised nutrition services based on DNA than for personalised nutrition services that look at only everyday eating habits’. And, instead of using the word ‘can’, use the word ‘may’. This includes other places (such as L 385) where you use the word ‘can’, it should be replaced with ‘may’.

L 368 (p 19) advice (not advices, which is not a word) is both singular and plural

L 369 (p 19): instead of writing ‘have to do with’, may I suggest, ‘are related to’…

L 371-372 (p 19) I do not understand what you are attempting to say with this sentence: ‘consumers may look up to the efforts that come with measuring yourself and filling out questionnaires’… Also, just a small note, questionnaires are not ‘filled out’ they are filled in… ‘filling out’ a questionnaire is a colloquialism used in spoken language.

L 376 (p 20) ‘Onwezen et al. talk about’, ‘discuss’ is a better scientific word.

L 380 (p 20) ‘but also different than emotions’, do you mean instead to write, ‘but also different from emotions’?

L 385-390 (p 20) pay attention to your run-on sentences.

L 391 (p 20) ‘to distinguish different types’ should be written as, ‘to distinguish between different types’…

L 395 (p 20) be careful how you phrase your suggestions about what future research ‘should do’, may I suggest instead writing, ‘It would be beneficial for future research to explore’…

CONCLUSION:

L 402 (p 21) may I suggest you remove the word ‘obviously’ as it is a polarising word and has the potential to make the reader look or feel stupid.

L 403 (p 21) instead of writing ‘per person’, a more scientific way is to write ‘in individuals’…

L 407 (p 21) instead of writing ‘someone’s eating context’, a more scientific expression would be, ‘taking individuals’ eating context’…

I am finished with my comments, but I do have a few questions: How can a policy or an incentive take into account an individual’s eating context? What is an example of what a policy should employ? Or what incentive should be employed? If you can list examples of some ideas you may have, then it offers a clearer picture of what can be done to improve areas that may need improving, in terms of trying to improve public health or personal health.

Also, what specifically does personalise nutrition advice based on DNA profiling mean? What is required and how can individuals, who may be interested in participating, be assured that their DNA profiling will not be abused or used by governments who may desire to gather ‘profiles’ of certain people to use for whatever they may want to use DNA profiling for. Or companies that may try targeting susceptible profiles, how are these individuals protected. What sort of data protection is there for these personalised nutrition services?

Personally, I find it a little scary that your research was funded by an agriculture and food company because that suggests they are interested in profiling people, which by default, I do not trust food and agricultural companies.
