# Supplementary material for: Consumer acceptance of personalised nutrition: The role of ambivalent feelings and eating context
Source: PLoS One. 2020 Apr 13;15(4):e0231342. doi: 10.1371/journal.pone.0231342 (PMC7153894; doi:10.1371/journal.pone.0231342)
Supplement: Attachment — Submitted filename: PONE-D-19-26890_Rebuttal revision.docx [file pone.0231342.s005.docx]

Ms. Ref. No.: PONE-D-19-26890

Title: Consumer acceptance of personalised nutrition: The role of ambivalent feelings and eating context

Journal: PLOS ONE

# EDITOR'S comments/ JOURNAL REQUIREMENTS:

Thank you very much for the constructive and helpful reviews and for giving us the opportunity to revise our manuscript. We substantially revised the manuscript based on the comments. Both a revised manuscript with track changes as well as a ‘clean’ version are resubmitted to the journal.

Journal Requirements:

1. When submitting your revision, we need you to address these additional requirements.

Please ensure that your manuscript meets PLOS ONE's style requirements, including those for file naming. The PLOS ONE style templates can be found at

<http://www.journals.plos.org/plosone/s/file?id=wjVg/PLOSOne_formatting_sample_main_body.pdf> and <http://www.journals.plos.org/plosone/s/file?id=ba62/PLOSOne_formatting_sample_title_authors_affiliations.pdf>

We checked PLOS ONE’s style requirements and adapted the manuscript accordingly. We also adjusted the references to the reference style of PLOS ONE. Furthermore, to make sure that the revised manuscript is without language errors, we copyedited the manuscript by a professional language-editing service.

2. Please include captions for your Supporting Information files at the end of your manuscript, and update any in-text citations to match accordingly. Please see our Supporting Information guidelines for more information: <http://journals.plos.org/plosone/s/supporting-information>.

Thank you, captions for the Supporting Information files are now included at the end of your manuscript. In total, we have three Supporting Information files: S1 (Original questionnaire in Dutch), S2 (Translated questionnaire in English) and S3 (Raw SPSS datafile). In addition, where relevant we refer in-text to these Supporting Information files.

3. Please include additional information regarding the survey or questionnaire used in the study and ensure that you have provided sufficient details that others could replicate the analyses. For instance, if you developed a questionnaire as part of this study and it is not under a copyright more restrictive than CC-BY, please include a copy, in both the original language and English, as Supporting Information. Moreover, please include more details on how the questionnaire was pre-tested, and whether it was validated.

We added a copy of the questionnaire, in both the original language and English, as Supporting Information. Furthermore, next to the information that was already described in the manuscript, we added the following information about pre-testing of the questionnaire (lines 161-166*):

*“Before the data collection was started, researchers pre-tested several versions of the questionnaire. Pre-tests of early versions of the questionnaire focussed on whether the questions and items were understandable and well-formulated. Different online versions of the questionnaire were then pre-tested to detect any final errors and to test the length of time that it took to complete the questionnaire. The study was approved by the Social Sciences Ethics Committee of Wageningen University & Research in the Netherlands.”*

*Note that in our rebuttal we refer to the line numbers of the ‘clean’, unmarked version of the manuscript.

4. We noticed you have some minor occurrence of overlapping text with your following previous publication(s), which needs to be addressed:

- Onwezen, Marleen C., Machiel J. Reinders, and Siet J. Sijtsema. "Understanding intentions to purchase bio-based products: The role of subjective ambivalence." Journal of Environmental Psychology 52 (2017): 26-36.

The text that needs to be addressed involves some sentences of the Discussion.

In your revision ensure you cite all your sources (including your own works), and quote or rephrase any duplicated text outside the methods section. Further consideration is dependent on these concerns being addressed.

Thank you for drawing our attention to this carelessness. Although the mentioned duplicated text is relevant in the light of our findings, it was not our intention to just copy-paste this from a previous publication. We therefore reformulated the relevant text into (lines 361-370):

“*These findings suggest that conflicting evaluations of personalisation benefits and privacy risks may result in a hesitancy regarding the concept of personalised nutrition (e.g. is it beneficial or risky?), which decreases one’s adoption intention. Furthermore, these findings are in line with a relatively novel body of studies in which ambivalent feelings are also highly relevant in shaping consumers’ intentions towards novel products or concepts [46]. According to these studies ambivalence may be experienced as an emotional state, which may be used as information regarding the specific object [47], which is in our case personalised nutrition. This observation also corresponds with the feeling-as-information theory, which argues that individuals use their feelings as a source of information in behavioural responses [48].”*

Additionally, we once more checked the whole paper on possible duplicated text and rephrased sentences when necessary. In the new version of the manuscript there is no overlapping/ duplicated text anymore.

# Reviewers' comments:

Thank you very much for the constructive and helpful reviews and for giving us the opportunity to revise our manuscript.

## Reviewer #1:

My main comments are with regard to English and grammar – noted below. Otherwise, the manuscript is mainly very well written and the suggested revisions are minor.

Thank you for the kind words on the paper and for providing constructive feedback on English and grammar. Based on your suggestions we have corrected these errors (see responses below).

• Abstract, line 12 – check use of apostrophe for ‘individuals’

• Abstract, line 22 – I think ‘inversely’ might be a more appropriate word than ‘reversely’.

• Abstract, line 25 - ‘In conclusion’ would be better than ‘In sum’.

Thank you, we corrected these grammar errors. Note that with regard to the apostrophe for individual(s) we meant the singular form: individual’s (as in: the choice of an individual). We added -an- (an individual’s ...), so that it is clear what is meant.

• Introduction, line 42 – I cannot see Brug et al (1999) in the reference list.

Thank you for your alertness. You are right, we omitted to add this reference in the reference list, but we now added it to the list:

Brug, J., Campbell, M. & van Assema, P. (1999). The application and impact of computer-generated personalized nutrition education: A review of the literature. *Patient Education and Counseling*, *36*(2), 145.

• Introduction, line 43 – I think the authors need a reference to support the statement that more personally relevant nutrition information will lead to higher compliance. Otherwise, they could state ‘and may in turn lead to greater compliance’.

We thank the reviewer for pointing this out. Although an increasing amount of empirical studies is published in the past few years with positive indications that personalised dietary approaches may result in improved dietary behaviors and health (see for example Adams et al., 2019), none of these studies explicitly focused on compliance. Thus we changed the sentence as you suggested to ‘and may in turn lead to greater compliance than generic dietary advice’.

• Introduction, line 45 – check use of apostrophe in ‘individuals’

Thank you. The apostrophe was indeed wrongly used here, since we meant the singular form: individual’s.

• Introduction, line 50 – is a reference needed to support the statement that there is a high penetration rate of smartphone ownership? Also, perhaps ‘high prevalence’ is a better phrase than ‘high penetration rate’?

We added references to support this statement: *‘[...], and the high penetration prevalence rate of smartphone ownership (**up to 40% worldwide and 87% in Dutch households respectively (Statistica, 2018; CBS, 2018))’*

• Introduction, line 52 – check sentence structure. I think it needs to read ‘(e.g. smart wearables) are becoming rapidly available’.

Thank you for pointing us to this language use. We adopted reviewer’s suggestion.

• Introduction, line 53 - a reference is needed to support the statement that consumer interest in these tools is growing.

We added the following reference: Smith, A. (2017). *Record shares of Americans now own smartphones, have home broadband*. Pew Research Center, 12 January 2017. <https://www.pewresearch.org/fact-tank/2017/01/12/evolution-of-technology/>

• Introduction, line 99 – check use of apostrophe in ‘consumers’.

• Introduction, line 110 – ‘a’ is not needed, i.e. ‘adhering to personalised advice’.

• Introduction, line 111 – ‘advice’ is the plural.

• Introduction, line 111 – no comma needed after ‘preferences’.

• Introduction, line 115 - check use of apostrophe in ‘consumers’.

Thank you, we corrected these grammar errors. The apostrophe was indeed wrongly used here, since here we meant to use the plural form: consumers’.

• Introduction, lines 118-122 – I think this is better in the Discussion, and the Introduction should end with the aim of the study.

The reviewer is right and some of these sentences were already in the Discussion section. We now (partly) moved the sentences to the Conclusion section of the manuscript.

• Methods, line 130 – ‘voluntariness’ is not a word.

We thank the reviewer for pointing this out and rephrased the sentence as follows: *“At the start of the questionnaire potential respondents received information about the study in which was explained that participation is on a voluntary basis and data is treated anonymously.”*

• Methods, lines 146-147 – please explain what is meant by low, moderate and high education level.

We used the Dutch education system and categorized this into low, moderate and high education levels using the international conventions. More details can be found in the added original and translated questionnaires (see Supporting Information). See also the table below for a more detailed explanation of the education levels. For reasons of comparison we also added the British education system:

| **Level** | **Dutch education system** | **British education system** |
| --- | --- | --- |
| Low | - LO (Primary school) - LBO (Lower vocational education) - Lower secondary education and pre-vocational secondary education (VMBO, MAVO, 3jr VWO, 3jr HAVO) | - No school - Primary - Some secondary school - ‘O’ levels/CSE/GCSE |
| Medium | - MBO (Vocational Education) - Higher secondary education (HAVO, VWO, Atheneum, Gymnasium) | - ‘A’ levels/HND/Highers - Technical/trade studies/GNVQ (incomplete) - Technical/trade studies/GNVQ (complete) - Some university/polytechnic (incomplete) |
| High | - HBO (University of Professional Education) - WO (University, polytechnic) - Postgraduate degree | - University/polytechnic graduate - Some postgraduate studies - Postgraduate degree |

In the text, we now added a few sentences to further explain the categorization low, moderate and high (and for more details we now refer to the questionnaire which is added in the Supporting Information) (lines 139-144):

*“Generally, in the Dutch education system, low education indicates only primary school and lower secondary education, moderate education indicates higher secondary education and vocational education, and high education includes those with university, postgraduate degrees, and professional education. For more details about education levels, refer to the questionnaires (both original and translated versions) in Supporting Information parts S1 and S2.”*

• Table 1 – please give SD for age as well as the range.

The SD (15.88 years) as well as the range (18 to 75 years) was added in Table 1.

• Methods, line 194 – please explain why means and SD are presented, yet Spearman’s rank correlation was performed. It is more usual to see medians presented if using Spearman’s correlations.

We thank the reviewer for pointing us to this error. In fact, we performed the right correlations (as shown in Table 3), but mentioned the wrong name in the text. So, instead of using Spearman’s rank correlation, we indeed used Pearson correlation coefficients, which is a measure of the linear correlation between two variables and represents the covariance of these two variables divided by the product of their standard deviations. We corrected this error in the text.

• Table 4 – it would be useful for abbreviations to be explained in footnotes to the table.

We now added the abbreviations that are used in the table in the footnote of the table.

• Discussion – please discuss the generalisability of your findings, i.e. are the consumer panels considered representative of the wider population?

A discussion on the generalizability of the findings has been added in lines 445-456:

*“An additional limitation could relate to the generalisability of the results to other countries. The sample that has been used is representative of the adult Dutch population in terms of gender, age, level of education and income. Future research should replicate the study in other countries to judge the generalisability of the findings. Nevertheless, the observation that our findings are in line with Berezowska et al. and Stewart-Knox et al. [22,33], who included eight or nine European countries in their studies, makes us confident that our findings are at least typical of other European countries. Berezowska et al. state that their cognitive model, which includes personalisation benefits, perceived privacy risks and the privacy calculus is robust and applicable throughout Europe [22]. Stewart-Knox et al. state that the Netherlands rated eating context as a significantly lower barrier than did other European countries [33], which may suggest that the importance of eating context will be even more important when our model is tested in other European countries.”*

• Discussion, line 315 – please check use of apostrophe in ‘consumers’ – consumers’?

• Discussion, line 368 – ‘advices’ is incorrect. Suggest rephrase to ‘(fictional) examples of nutritional advice’.

Thank you, we corrected these grammar errors. The apostrophe was indeed wrongly used here, since here we meant to use the plural form: consumers’.

## Reviewer #2:

Overall, this concept of ‘Personalised Nutrition Services’ sounds interesting. It may have potential to influence some individuals who are interested in these types of apps or technology.

We would like to thank the reviewer very much for his/her constructive criticism and helpful feedback.

However, the biggest issue I have with this article is the questionnaire. I do not understand exactly how the questions are phrased. I feel it is important that the authors include the original questionnaire they used for the survey if the reader is to follow the authors’ explanations and results. Specifically because the authors discuss (p 10) the 7-point Likert scale of the questionnaire ranging from 1: strongly disagree to 7: strongly agree. But then in Table 2, for example, scale number 3 ‘Ambivalent feelings’ has three items: ‘I feel no conflict at all/I feel maximum conflict’. From the way the authors present their work, I cannot determine how a participant’s response to this question is to be interpreted. In other words, does the participant ‘strongly agree or disagree’ with ‘feeling no conflict or maximum conflict’ what does or how is the respondent’s answer interpreted? Another way of stating my question is: Is the Likert scale response 1: ‘strongly disagree’ to 7: ‘strongly agree’, or is it, 1: ‘I feel no conflict at all’ to 7: I feel maximum conflict’? Including the original questionnaire may help to clarify the answer to this question.

We thank the reviewer for addressing this unclarity. To answer the question of the reviewer how a participant’s response to the different questions should be interpreted: indeed most questions were formulated as statements where respondents had to indicate to what extent they agree on a Likert-type scale ranging from 1 (‘strongly disagree’) to 7 (‘strongly agree’). However, ambivalent feelings were measured on a somewhat different answer scale (called a semantic differential), where respondents had to indicate their feelings ranging from 1 (e.g., ‘I feel no conflict at all’) to 7 (e.g., ‘I feel maximum conflict’). The same applied for the risk-benefit calculus (i.e., 1= greater risks to 7 = greater benefits). All other variables are measured as Likert-type scales. To make this more clear, we not only added a copy of the questionnaire, in both the original language and English, as Supporting Information in the revised version of the manuscript, but now also more explicitly mention this in the ‘2.2 Measures’ section (see also our response to the next comment).

In addition, p 10 should be written more succinctly. The authors need only to state once that all scales are based on a seven-point Likert scale response, and then describe each question or scale. As an example lines 175-176 would be clearer if the authors wrote something more similar along the lines of: “Privacy Risk was measured using 3-items which explored issues around privacy and privacy-related risks, and was based on Berezowska” etc. In trying to explain this questionnaire, it might serve the authors better if they include the questionnaire in a box or table and then discuss it that way.

We agree with the reviewer that the ‘2.2 Measures’ section could be written more succinctly. We therefore rewrote this paragraph in a similar way as suggested by the reviewer. To further increase the readability of this section, we now immediately (at the beginning of this section) refer to Table 2, where the formulated items can be found. Additionally, we refer to the complete original and translated questionnaires that are now included in the Supporting Information S1 and S2.

Moreover, all responses, 1 through 7, should be listed as to what each response equates to, because I do not know what a response of 3 or 4 equates to (ex. L 217, p 13) The authors need to be clear about what the ‘mean’ suggests, in terms of understanding how to interpret these scales accurately.

We added a copy of the questionnaire, in both the original language and English, as Supporting Information in the revised version of the manuscript, to make it more insightful how the recorded responses looked like for the respondents. It is common in this type of surveys to name the endpoints of the answering scale (in this case the 1 and 7), with 4 as the (neutral) midpoint. In terms of understanding how to interpret these answers on the scales, note that the results section has been extended with a more detailed explanation of our findings. See also our reply to the specific comments below, under the headings ‘RESULTS’ and ‘STRUCTURAL MODELS ESTIMATION’.

The authors need to be clearer on how the results are interpreted. In other words, it is not enough to report ‘our results show’. Results need to be explained.

We thank the reviewer for addressing this unclarity. In the revised version of the manuscript, the results section has been extended with a more detailed explanation of our findings. See also our reply to the specific comments below, under the headings ‘RESULTS’ and ‘STRUCTURAL MODELS ESTIMATION’.

ABSTRACT:

- L 12: The first line of the abstract the author uses the word ‘can’ contribute to the prevention of non-communicable diseases… can is a strong word, is there proof of this? I suggest using the word ‘may’ unless you have strong scientific proof where you can back it up with references.
- L 25: …the current study ‘shows’… this current study can only ‘suggest’, as it takes proof to ‘show’ that there is a connection between one thing and another thing.
- L 28: instead of using the word “someone’s”, may I suggest that you use “individual’s”, and this would be the case throughout the rest of the article when you use ‘someone’s, as this expression is not scientific.

Thank you for pointing us to this language use. We adopted reviewer’s suggestions. Note that we replaced ‘someone’ throughout the paper with ‘individual’.

INTRODUCTION:

- L 34 (p. 3), instead of ‘needs by so-called’ it is preferable to express it as ‘needs by what have been termed ‘personalised nutrition services’ …and using the brackets to demarcate the term. Also, you expressed that it has ‘great’ potential, is this exactly what your reference Celis-Morales et al. 2015 expressed? Or is that your opinion? I would be careful about using emotive expressions because they are like promises you cannot possibly keep.

Thank you for this comment. We adopted the reviewer’s suggestion regarding the formulation. In addition, regarding Celis-Morales et al. (2015), they expressed the following in their paper: “Evidence-based, personalised interventions which incorporate effective BCT and which are delivered digitally are likely to be an important route to scalable and sustainable interventions.” We agree that ‘great’ is not suitable in this case and deleted the word. Instead, we also added Adams et al. (2019) as reference, as these authors argue that “personalized approaches may result in quantifiable improvements in dietary behaviors and health outcomes compared with conventional approaches.” (p. 1)

- L 37 (p. 3) ‘obesity have not been very successful’… does the WHO 2011 reference actually report this? I suggest finding a more scientific way of reporting this, such as: ‘One-size-fits-all attempts to turn the tide of obesity have not been successful at reducing the prevalence of obesity’ (or something along those lines). Again, it is just removing the strong emotive words, or opinionated or watered-down words.

Thank you for this comment. The WHO 2011 report states the following: “*In addressing noncommunicable diseases, the population-wide approach to prevention described in the previous chapter has great potential to decrease disease burden, but it does not provide an adequate response to the need to strengthen health care for people with NCDs (i.e., non-communicable diseases). The disease burden can be reduced considerably in the short- to medium-term if the population-wide approach is complemented by health care interventions for individuals who either already have NCDs or those who are at high risk.*” And in the final chapter, the report concludes “*Both population-wide primary prevention approaches and individual health-care strategies are needed to reduce NCDs and their impact.*” So, the authors of this WHO report say that although population-wide approaches have their merits, they need to be complemented with more individualized approaches. Additionally, Fallaize et al. (2013) state that (p. 40): “*Whilst population guidelines go some way to account for this, using characteristics such as age and sex, an increased understanding of the role of genetics in nutrient metabolism has highlighted the inadequacy of this ‘one-size-fits-all’ approach.*”

Taken together, we reformulated the first sentences of the paper into (lines 35-40):

“*The incidence of non-communicable dietary related diseases such as type-2 diabetes, and cardiovascular disease is growing (WHO, 2014). In this respect, Fallaize et al. (2013) reported the insufficiency of a ‘one-size-fits-all’ approach to dietary guidance. Instead, dietary interventions tailored to individuals’ personal nutritional needs by what have been termed ‘personalised nutrition services’ show the potential to improve healthy dietary patterns and public health (Adams et al., 2019; Celis-Morales, Lara, & Mathers, 2015).”*

NB. Note that we updated the WHO 2011 reference with the WHO Global status report on noncommunicable diseases 2014.

- L 39 (p 3) ‘personalized’ is correct but you need to be consistent in all your spellings. In other words, you should spell it as ‘personalised’ since that is generally how you spell it throughout your paper.

The reviewer is correct that we used both the words ‘personalized’ and ‘personalised’. We now changed it to ‘personalised’ (British spelling) throughout the paper.

- L 41 (p 3) ‘Personalisation will make’… it is better to express it as ‘Personalisation has the potential to make the provided’…
- L 45 (p 3) instead of using the word ‘like’ it is better to say ‘such as’

Thank you for pointing us to this language use. We adopted reviewer’s suggestions.

- L 47 – L51 (p 3) is a run-on sentence, break it up into at least 2 sentences. Also, do you have a reference for the sentences of Lines 47 through 53? Otherwise, it sounds like it is your opinion that consumer interest is growing. How do you know consumer interest is growing?

Thank you for this comment. We agree that in the mentioned lines we made statements that we ourselves took for granted without referring to the literature. We now added references (see also our response to the comment made by Reviewer #1).

- L 54 (p 3) do you have a reference about ‘personalised nutrition services’ as having ‘great potential’ and ‘consumer reluctance’, both these need to be supported by a reference.

Thank you for this comment. As mentioned in a previous comment, we agree that ‘great’ is not suitable in this case and deleted the word. Furthermore, a reference for consumer reluctance to use personalised nutrition services is added. The study referred to (Wendel, Dellaert, Ronteltap & van Trijp, 2013) examined consumers’ intention to use health recommendation systems to receive personalised nutrition advice.

Added reference:

Wendel, S., Dellaert, B.G., Ronteltap, A., & Van Trijp, H.C. (2013). Consumers’ intention to use health recommendation systems to receive personalized nutrition advice. *BMC Health Services Research*, 13(1), 126.

- L 55 (p 3) ‘crucial’ sounds a bit too strong, my suggestion is ‘it would be advantageous to understand’… Also, what are the references for consumers’ lack of knowledge about these devices or apps? Doesn’t that play a role? Consumer reluctance implies that ‘all consumers’ are aware of these devices or apps but are reluctant to use them.

Thank you for this comment. We changed the word ‘crucial’ as it is indeed too strong. Furthermore, we agree that lack of knowledge or awareness about devices and apps that deliver such personalised services would be an important aspect in the uptake of these type of services, but literature more often emphasized the reluctance of consumers to use personalised recommendation systems due to a lack of trust or perceived benefits (see also Wendel et al., the reference in the previous comment). Also, the sentence referred to has to be seen in the context of the previous sentences in which we state that awareness is rising (i.e., “technological do-it-yourself tools for health measurement and monitoring (e.g. smart wearables) are becoming rapidly available, and consumer interest in these mHealth tools is growing”). So, we argue that given this growing awareness of the technological possibilities, consumers are still reticent to use personalised nutrition services.

- L 61 & 64 & 78, 109 etc (p 4) same comment as expressed earlier in L 25, it is hard to prove that a study ‘shows’ anything, at best, a study suggests or may indicate… this comment is the same for other lines where you use the word ‘show’.

Thank you for pointing us to this language use. We adopted reviewer’s suggestion to replace the word ‘show’ where necessary (i.e. not in cases like ‘This table shows ...’ where factual information is presented).

- L 62 (p 4) ‘and the less the perceived risks’… there are possibly too many ‘thes’ in this sentence, I’d remove the second ‘the’, or technically the fourth ‘the’ in that sentence.
- L 63 (p 4) I think you mean ‘the greater is the consumers’ intention to adopt’ (as this reads better or more clearly)

Thank you for pointing us to this language use. We adopted reviewer’s suggestions.

- L 64-67 I’m not certain I understand this sentence. Do you mean that individuals are not willing to enrol in personalised nutrition services because they feel they are putting their privacy at risk? If this is the case, I would take these words ‘and putting at risk their privacy’ out of the parentheses and re-word the sentence. I suggest something along the line of: ‘Consumers feel that disclosing their privacy information is too big of a risk compared to the benefits that personalised nutrition services can offer’.

This is indeed what is meant with this sentence. The sentence is now re-worded into: *“For example, Berezowska et al. suggested that consumers feel that disclosing their privacy information is too great a risk compared to the benefits offered by personalised nutrition services [22].”*

- L 68 (p 4) instead of saying ‘they call’ say ‘they have termed it’, or ‘which has been termed’. Also L67-71 is a run-on sentence. Personally, I would place a period after the parentheses and start a new sentence with ‘This Privacy Calculus is…’
- L 71-75 is a run-on sentence… I do not understand this sentence, I think it’s because you’re trying to say too much in one sentence. Break up in individual sentences what it is you’re trying to convey.

Thank you for pointing us to this language use. We adopted reviewer’s suggestions. L71-75 is rephrased into: “Note that the perceived benefits of personalised nutrition services are intrinsically associated with the *concept* of personalised nutrition—that is, the anticipated individual health and fitness benefits receiving from personalised dietary recommendations. On the other hand, perceived risks reflect consumers’ concerns associated with the *delivery system* of personalised nutrition advice—that is, concerns about the handling of privacy-sensitive information (Stewart-Knox et al., 2013).”

Additionally, to put emphasis on where the difference between benefits and risks lies, we have italicized the relevant words (i.e. concept and delivery system).

- L 95 (p 5) ‘is perceived to have both benefits’ do you mean factors? Is having food neophobia a benefit, or food neophilia a benefit. (I know I am knit-picking here, but your choice of words has to be concise because words convey an image and meaning, and I’m not sure that either food neophobia or neophilia can be seen as ‘benefits’, I see them as factors that have to be considered when making these personalised nutrition apps or devices, in order to ‘personalise’ nutrition suggestions.

Thank you. We tried to clarify this and now rephrased this sentence into: *“As personalised nutrition may simultaneously stimulate both the urge towards a better health and fear of data misuse, ambivalent feelings among consumers may very well be the result (Van Trijp & van Kleef, 2008).”*

- L 99 & 115 (p 5 & 6) should be written as: consumers’ adoption
- L 110-111 (p 6) ‘in adhering to a personalised advice’, remove ‘a’; ‘eating at other people’s houses’ instead perhaps say it as ‘eating in friends’ or acquaintances’ homes. Also, remove the s on advices. Advice can suggest both the singular and plural. Instead of ‘ideally take the preferences’, should instead perhaps be written as: ‘would benefit from considering the eating preferences of others in the eating context over the preference of the individual’.
- My own comment on the above sentence is that it renders ‘personalised nutrition services’ as no longer being ‘personalised’. Perhaps there should be an app or device that is called something different in order to service these needs, such as ‘group or context nutrition services’…
- L 116-117 (p 6) sentence might be better if written as: ‘to build on previous research, (which has investigated personalised nutrition advice), by exploring’…
- L 118-119 (p 6) this sentence would be crisper if you added ‘factors help to further explain’… ‘Additionally, our work sheds light into how these factors may relate’…
- L 121 (p 6) instead of saying ‘article’, perhaps work or research is a better expression, also findings ‘may potentially’ be used to design personalised…

Again, we thank the reviewer for showing the correct language use and incorporated reviewer’s suggestions. As English is not our native tongue, we’re grateful for this constructive suggestions for improvement!

- METHODS section (p 6 & 7) needs to be written more scientifically. Also, it would be helpful if the authors state exactly what a response of 4 equates to (L 142).

We critically looked at the Methods section, and rewrote parts of it when necessary (see also our responses to the next comments). With regard to the question what a response of 4 equates to (L142), this equates to the midpoint of the scale in case of a scale ranging from 1 (not at all) to 7 (totally). We rewrote the relevant part of the text, to make this more clear (lines 130-134): “*[...] to ensure that participants understood the provided definition of personalised nutrition, we asked them to rate on a scale ranging from 1 (not at all) to 7 (totally) whether they had a clear picture of personalised nutrition advice. Only those who provided a score equal to or higher than the midpoint of the scale (4 or higher), were eligible for the study.*”

- L 144 (p 7) you state that 797 respondents comprise your sample, but your abstract states that your data was from 996 (L 17, p 2) respondents?

The 996 respondents was the original sample, but the final sample consists of 797 respondents after removing the participants that either indicated that they already use some form of personalised nutrition or indicated that they don’t have a clear picture of what personalised nutrition advice is. But we acknowledge that this may be a bit confusing, therefore we now report in the abstract also the final sample size of 797 participants.

- L 146 (p 7) what is ‘low, moderate and high’ education?

See also our response to Reviewer #1, who made a similar remark:

We used the Dutch education system and categorized this into low, moderate and high education levels using the international conventions. More details can be found in the added original and translated questionnaires (see Supporting Information). See also the table below for a more detailed explanation of the education levels. For reasons of comparison we also added the British education system:

| **Level** | **Dutch education system** | **British education system** |
| --- | --- | --- |
| Low | - LO (Primary school) - LBO (Lower vocational education) - Lower secondary education and pre-vocational secondary education (VMBO, MAVO, 3jr VWO, 3jr HAVO) | - No school - Primary - Some secondary school - ‘O’ levels/CSE/GCSE |
| Medium | - MBO (Vocational Education) - Higher secondary education (HAVO, VWO, Atheneum, Gymnasium) | - ‘A’ levels/HND/Highers - Technical/trade studies/GNVQ (incomplete) - Technical/trade studies/GNVQ (complete) - Some university/polytechnic (incomplete) |
| High | - HBO (University of Professional Education) - WO (University, polytechnic) - Postgraduate degree | - University/polytechnic graduate - Some postgraduate studies - Postgraduate degree |

In the text, we now added a few sentences to further explain the categorization low, moderate and high (and for more details we now refer to the questionnaire which is added in the Supporting Information) (lines 139-144):

*“Generally, in the Dutch education system, low education indicates only primary school and lower secondary education, moderate education indicates higher secondary education and vocational education, and high education includes those with university, postgraduate degrees, and professional education. For more details about education levels, refer to the questionnaires (both original and translated versions) in Supporting Information parts S1 and S2.”*

- The box on page 9 needs a legend. Additionally, the example in the box for ‘Personalised nutrition advice’ seems very generic as well. Perhaps not as generic as prescribing eating whole grain products. But the ‘Personalised nutrition advice to eat yoghurt with oatmeal instead’ could be perceived by respondents as too generic and not personal to them. In my opinion, personalised nutrition advice should be specifically making suggestions about how many grams of fibre and/or calcium or vitamin D and B vitamins, magnesium, etc., a day an individual is getting (based on the example here of why a person should choose oatmeal and yoghurt since it contains all these micro-nutrients). And again, it is just my opinion, based on this example, that my first impression is that I see ‘personalised nutrition advice’ as a gimmick, and my gut tells me not to trust it. However, from your questionnaire you have no way of knowing if any of your respondents may have felt this way as well. And, as a result, I think you need to be very careful how you interpret your results. Furthermore, you should not use expressions such as, ‘our results show or imply’ (as in L220, p 13) because again, your results cannot show, they can only suggest what you think the interpretation is...

A legend was added to the text box on p. 9. Furthermore, we greatly appreciate reviewers’ cautionary note. Personalised nutrition advice can indeed take on different forms, from very specific advice to more generic, abstract advice. In this case we used the more generic advice for several reasons such as that we could not truly personalize the advice in this experiment (i.e., to provide specific advice we would need much more information from the user) and because we want to examine a more general response to the personalised nutrition concept instead of responses to specific types of personalised advice. But the reviewer is right that we have to mention this as a shortcoming of the study. We now added this as a limitation to the Limitations-section of the revised manuscript (lines 420-434):

*“A major limitation of this study is that only one definition of personalised nutrition services is used in the research: personalised nutrition is based on personal preferences and/or self-formulated goals and/or eating habits and/or health status and/or DNA. This description of personalised nutrition is very general; participants may have reacted differently to different examples of how personalised nutrition may look like. Consequently, it is possible that the perceived benefits and risks of personalised nutrition services may be different for those individuals who considered personalised nutrition services based on DNA as opposed to individuals who considered personalised nutrition services that only take into account everyday eating habits. Furthermore, the examples that were provided to illustrate the definition could be perceived by respondents as too generic and not personally relevant. This may have led consumers to view the concept of personalised nutrition as a gimmick. Since our study did not control whether participants may have felt this way, our findings need to be interpreted carefully. Future research may refine the insights obtained in the current study by including a variety of more specific (fictional) nutritional advices to further examine how individuals react to different types of personalised nutrition recommendations..”*

DATA ANALYSIS:

- Page 12 I do not feel I have enough experience in this respect to make any comments, the only question or comment I can make with respect to this section is: Did you carry out a normalcy distribution test on your data? I do not think you can assume that your data is normally distributed, as is suggested by reporting the mean and standard deviation. If your data is not normally distributed, how can you know that mean and standard deviation is the best way to present your data? As median and interquartile range may be a better way to present it. It might change the results of your findings. Also, looking at ‘mode’ can also be a different way to investigate your data. By looking at just the average (or the mean), the average has a way of watering down or equalising what you might be finding. You might be missing important details if you are looking at just one data point (as in, looking only at the mean).

We thank the reviewer for providing this critical note on how our data is distributed. The reviewer is right in that we cannot assume that our data is normally distributed, as in our study, we mainly use 7-point Likert-type scales to measure participants’ opinion on several aspects (variables) related to personalised nutrition. Using this type of non-continuous, ordinal data is quite common practice in the behavioural sciences. Nevertheless, while this data is by definition not normally distributed, there are different views on the treatment of Likert-type data. Statisticians usually treat it as ordinal data, but among social scientists it is a convention that when you have a big sample size* and data from multiple Likert-scale questions is summed together (i.e., multi-item scales, which have to be checked on internal reliability), this sum can be treated as interval data that can be analysed through parametric statistics, e.g. mean and standard deviation can be calculated. There are numerous examples available in literature that support this (for example, see for an overview of multi-item scales with empirical evidence of their psychometric qualities *Marketing Scales Handbook* by Gordon Bruner II).

Note that, as an additional check, we also examined the bivariate correlations by using bootstrapping, an estimation technique which does not assume normally distributed data. For each of the significant correlations, the 95% confidence intervals that resulted from the bootstrapping estimation technique did not intersect with zero, which implies that there indeed were statistically significant effects.

* Because of the central limit theorem, which argues that there are a variety of situations in which normality can be assumed regardless of the shape of sample data, we do not really need to worry about the assumption of normality in larger samples (see Field, 2013)

References:

Bruner II, G.C. (2015). *Marketing Scales Handbook: Multi-Item Measures for Consumer Insight Research (Volume 10)*. Fort Worth, TX: GCBII Productions, LLC.

Field, A.P. (2013). *Discovering statistics using IBM SPSS Statistics: And sex and drugs and rock ‘n’ roll* (4^th^ ed.). London: Sage.

RESULTS:

- L 216 (p 13), you write that ‘respondents provided the highest average scores’. Respondents did not ‘provide’, it is more accurate to report that ‘respondents had the highest mean score’…
- L 220 (p 13) again, your results cannot imply anything, but only suggest.

We thank the reviewer for offering these suggestions and improved the text.

- L 217 (p 13) what does a mean score of 5.16 suggest? How is a score of 4.88 interpreted exactly?

The reviewer is right that our explanation of Table 3 was very short and the explanation and interpretation of the values in the Table could be much better. We therefore substantially expanded this section with descriptive results and a discussion of the meaning of the mean scores on the different items have been added (lines 237-251):

*Participants provided the highest mean average score to Personalisation Benefit (M = 5.16). This score, which is well above the midpoint of the scale (which is a score of 4), suggests that participants perceive benefits to personalisation. The mean score on Privacy Risk (M = 3.47) is just below the scale midpoint, which suggests that participants perceive moderate risks associated with receiving personalised nutrition advice. These scores also suggest that participants perceive greater benefits than risks, which is also reflected in the mean score on Risk-Benefit Calculus (M = 4.88). This score, which is above the scale midpoint, suggests a positive balance towards more perceived benefits than perceived risks. The mean score on Ambivalent Feelings (M = 3.50) is just below the midpoint of the scale, suggesting that participants have moderately ambivalent feelings about using personalised nutrition advice. In contrast, the mean score on Eating Context Barrier (M = 4.43) is just above the scale midpoint, suggesting that participants perceive eating context as a potential barrier to using personalised nutrition advice. Finally, the mean score on Intention to Use Personalised Nutrition Advice (M = 3.86), which is just below the scale midpoint, suggests only a moderate intention to make use of personalised recommendations.*

STRUCTURAL MODELS ESTIMATION:

- L 234 -276 (pp 14-16) It would be helpful if you write exactly what it means when items are positively or negatively associated. Clearly explain what these correlations suggest. In other words, what does it mean when you write that ‘Personalisation Benefit was negatively related to Ambivalent Feelings? Does it suggest that as an individual’s ambivalent feelings increase their personalisation benefits decrease? And if this is the case, clearly state it. If the results are not clearly stated, it is my opinion that there is too much room for ambiguity in your results and you leave it to the reader to decide what the findings mean. Personally, I find myself getting lost in what the findings mean, and I don’t know that I necessarily trust the authors’ opinion on their results mainly because the structure and phrase-ology of the questionnaire is not clear. Listing each item’s mean and SD scores and explaining exactly how each score is interpreted, may help me as a reader to understand better how the questionnaire items can be understood. I find that I am confused about aspects related to, as an example, privacy risk and benefit: Do the respondents think that ‘personalised nutrition advice’ is a risk, or do they think it benefits them.

Thanks again for pointing out that the way in which we presented our results was unclear. This is also in line with your previous comment. In the revised version of the manuscript, the results section has been extended with more thorough explanation on the meaning of our findings throughout the section. Below you find some examples how we extended the interpretation of the results:

Lines 288-290: *“...suggesting that the intention to use personalised nutrition advice increases when the risk-benefit calculus is more positive, but decreases when participants experience more ambivalent feelings.”*

Lines 292-293: *“...suggesting that participants feel less ambivalent when more personalisation benefits are perceived.”*

Lines 295-296: *“...suggesting that participants experience more ambivalent feelings when more privacy risks are perceived.”*

Lines 298-301: *“In other words, perceived privacy risk (in contrast to perceived personalisation benefits) is more strongly related to the perception of ambivalent feelings, whereas the perception of personalisation benefits (in contrast to perceived privacy risks) is more strongly related to the risk-benefit calculus.”*

- L 278 (p 16) the figure is missing. However, I found it on the back of the last page, after the references. Not sure why it wasn’t where the figure legend is written.

We can understand the confusion, but the reason that the figure is missing, is that the journal guidelines prescribe that figures should be uploaded separately as individual files (which is apparently put at the back of the document when all files are converted into a pdf for revision). In the manuscript itself only the figure captions appear.

DISCUSSION: This section also needs to be written in better scientific language

- L 291 (p 16): ‘First, the current study confirms previous research’, using the word ‘confirms’ is not accurate. Your study may be in agreement with previous research, but it does not confirm it. Specifically, how did previous research take their measures? What were their methods? Did they use the same questionnaire as you used to take your measures?

Yes, we are able to replicate previous findings by using the same measurement scales as previous studies (i.e., Berezowska et al., 2015). This was also reported in the section 2.2 on ‘Measures’. So, given that previous research used the same questions as that we used to take our measures, we are able to replicate previous findings. We now state that our study is in agreement with previous research.

- Also, as I previously mentioned about your findings and when you discuss them, you have to be careful how you interpret what you have found. Because there may be other factors at play that you (or other researchers) have not considered or thought about, such as if individuals ‘think’ or ‘feel’ that personalised nutrition is a gimmick, or perhaps they feel it is too generic, or it just does not pertain to them. They may feel that personalised nutrition does not offer them anything new.

The reviewer is right that we only considered a limited number of aspects in our model, which make that we have to interpret our results with care. We now mention this explicitly as a major limitation of our study (lines 396-402):

*“First and foremost is that we only focused on a limited number of constructs that may play a role in consumers’ evaluation and usage intention of personalised nutrition services. There may be other factors that play a role in the evaluation of the concept of personalised nutrition that were not considered in this study, such as trust in different types of providers of these services (Poínhos et al., 2017; Wendel et al., 2013), one’s personal motivation and ability to change behaviour (Rankin et al., 2017), and the way or format with which personalised nutrition advice is communicated to consumers (Nguyen et al., 2017).”*

- Additionally, why did you not measure or consider those who know nothing about personalised nutrition? Wouldn’t it be advantageous to understand why this group have no knowledge of personalised nutrition? Isn’t this lack of knowledge another barrier for individuals accessing or using personalised nutrition apps or devices? And, on a similar vein, what about those who have used personalised nutrition advice? What were their thoughts and feelings regarding it? I think you have too conveniently brushed these participants aside. Although these two groups comprise fewer participants, it would be interesting to compare (and comment) on these outcomes as well.

We indeed did not explicitly ask people how much they know about personalised nutrition, we only asked whether people have an understanding of what personalised nutrition is based on the provided definition. So, we cannot separate the group of participants who know nothing about personalised nutrition. We agree with the reviewer that it would be interesting to understand why some people know more about the topic than others, but this was simply not within the scope of our research. Other barriers, that were not taken into account in this study, may play a role for people who lack knowledge of the concept.

Furthermore, we removed the small number of participants that have experience with some form of personalised nutrition from our sample. We agree that it would also be interesting to look at how these participants perceive benefits and risks, but the group was too small to conduct statistically sound analyses and compare it with the other group.

Nevertheless, we incorporate as a suggestion for future research that a distinction could be made in consumer groups with varying degrees of experience with the personalised nutrition concept (lines 463-472):

*[...], it would be interesting in future research to distinguish between different types of consumers or consumer groups. In this study we developed a generic model, but it is possible that the relations between the different concepts will work differently with diverse groups. For example, future research could distinguish between the degree of experience that various people have with the concept of personalised nutrition, meaning that different interventions, services or communications could be effective in varying degrees for different consumer groups. It would be beneficial for future research to explore how our results can be used effectively in real-life implementations of personalised nutrition services and whether it would be effective to differentiate between several consumer groups.*

- L 300-304 (p 17) I do not understand this sentence probably because I do not understand what you mean by ‘affective laden construct’. Can you describe what you mean by using that term? Also, your study can also suggest, not show. Also, you use the word ‘fact’ on occasion, and ‘fact’ is something you have to prove. Facts are hard to prove, so my suggestion is that you express it in terms of in Line 304 ‘These findings suggest that conflicting’…

First, with affective laden construct we mean that the construct of ambivalent feelings can be linked to affect/ emotions that are evoked by the concept of personalised nutrition. This in contrast with a cognitive construct like risk-benefit calculus, which is a rational/ cognitive assessment of benefits and risks. However, we agree that we could have better labelled it as an affective construct instead of affective laden construct, which we now did in the revised version. Furthermore, we once again thank the reviewer for the suggestions to improve scientific language and replaced the word ‘fact’ where necessary.

L 307 (p 17): ‘The fact that ambivalent feelings negatively predict usage intention indicates that ambivalence is experienced as a negative, aversive state’. I do not know that I can agree with this statement, particularly because I do not understand exactly how the questions in your questionnaire have been termed or how exactly they have been written. It would seem to me, that depending on how the questions were written, you should be able to untangle the exact feelings of each respondent. In other words, you should be able to separate out the individuals who have ‘positive’ feelings towards ‘personalised nutrition advice’ from individuals who have ‘negative’ feelings. It would seem to me that your data analysis would be better analysed if you allocate those participants with high scores from those with low scores, and then see what the correlation results are.

Maybe our formulation in the discussion section of the role of ambivalent feelings in the usage intention of personalised nutrition advice was not very clear. Based on our operationalisation of ambivalent feelings we are not able to just separate out the individuals who have ‘positive’ feelings towards personalised nutrition advice from individuals who have ‘negative’ feelings. Based on the scores on this construct it is only possible to distinguish between individuals that have more ambivalent feelings and individuals with less ambivalent feelings. See also the remark that we made in that regard in the ‘Limitations’-section (lines 410-416): *In addition, while ambivalent feelings have been included in the model, other positive or negative emotions were not. Onwezen et al. discuss the motivational function of ambivalence via emotions and argue that it remains unclear to what extent the effect of ambivalence comes from mixed feelings, or from positive versus negative emotions [46]. The authors conclude that, while ambivalence is a concept that differs from (the weighing of) perceived risks and benefits, it is also different from emotions. We did not control for the effect of emotions in this study, and can therefore not provide clarity in that respect.*

We reformulated the relevant text, also by referring to how ambivalent feelings were operationalised in the questionnaire, and tried to make it more understandable (lines 355-370):

*Second, this study extends previous research by suggesting the additional value of ambivalent feelings as an affective construct in predicting intention to use personalised nutrition services. Stated differently, feelings of conflict, uneasiness and mixed feelings evoked by the concept of personalised nutrition are negatively related to the usage intention of these types of advice. More specifically, this study indicates a negative association of ambivalent feelings with intentions, beyond the cognitive weighing of perceived benefits and risks by means of the risk-benefit calculus. These findings suggest that conflicting evaluations of personalisation benefits and privacy risks may result in a hesitancy regarding the concept of personalised nutrition (e.g. is it beneficial or risky?), which decreases one’s adoption intention. Furthermore, these findings are in line with a relatively novel body of studies in which ambivalent feelings are also highly relevant in shaping consumers’ intentions towards novel products or concepts (e.g. Onwezen et al., 2017). According to these studies ambivalence may be experienced as an emotional state, which may be used as information regarding the specific object (Priester & Petty, 1996), which is in our case personalised nutrition. This observation also corresponds with the feeling-as-information theory, which argues that individuals use their feelings as a source of information in behavioural responses (Schwarz, 2011).*

Furthermore, we removed the part of the text stating that individuals with more ambivalence perceive higher levels of risks and that ambivalence strengthens the association between risks and intentions, since by critical re-reading of this text we cannot argue that our results really support these claims that were made.

- L 328 (p 18): ‘important predictor of someone’s intention to use personalised nutrition advice. Besides an’ … instead should be written as: ‘important predictor of an individual’s intention to use personalised nutrition advice. Other than an’… and ‘few’ studies, as opposed to what you have written ‘not many’ studies.
- L 340 (p 18) & L 363 (p 19) spelling of individualized or individualised, and personalized or personalise, be consistent in your spellings.

Thank you for pointing us to this language use. We adopted reviewer’s suggestions.

- LIMITATIONS AND FUTURE RESEARCH: Needs to be written in better scientific language. As an example L350-351 (p 19): ‘A number of limitations exist in this study, and we include suggestions for future research. Firstly, self-reported measures were used to identify’…
- L 363-368 all need improving… additionally, I do not understand what you are saying when you write: L 363-364 (p 19): ‘This general description of personalised nutrition could have led to that respondents may have reacted to different “products”.

We completely rewrote this section 4.2 ‘Limitations and future research’. We not only improved language, but also incorporated additional limitations of the study (see our responses to different comments elsewhere in this document). By doing so, we hope that we addressed the above mentioned comments of the reviewer satisfactorily.

- I suggest writing L364-368: ‘As an example, it is possible that perceived risks may be more important for personalised nutrition services based on DNA than for personalised nutrition services that look at only everyday eating habits’. And, instead of using the word ‘can’, use the word ‘may’. This includes other places (such as L 385) where you use the word ‘can’, it should be replaced with ‘may’.

We adopted the suggestion and rewrote the text. We also replaced the words ‘can’ with ‘may’ throughout the paper, where necessary.

- L 368 (p 19) advice (not advices, which is not a word) is both singular and plural
- L 369 (p 19): instead of writing ‘have to do with’, may I suggest, ‘are related to’…

Thank you for pointing us to this language use. We adopted reviewer’s suggestions.

- L 371-372 (p 19) I do not understand what you are attempting to say with this sentence: ‘consumers may look up to the efforts that come with measuring yourself and filling out questionnaires’… Also, just a small note, questionnaires are not ‘filled out’ they are filled in… ‘filling out’ a questionnaire is a colloquialism used in spoken language.

What we wanted to say is that consumers may find it a lot of effort or too complicated to fill in the forms/ questionnaires that are needed to obtain personalised advice (i.e., containing health parameters and dietary intake measures). We adapted the text to make this more clear (lines 406-408): “*For example, consumers may find it too effortful or complex to conduct do-it-yourself health measurements and filling in questionnaires or food diaries in order to receive personalised advice.*”

- L 376 (p 20) ‘Onwezen et al. talk about’, ‘discuss’ is a better scientific word.
- L 380 (p 20) ‘but also different than emotions’, do you mean instead to write, ‘but also different from emotions’?
- L 391 (p 20) ‘to distinguish different types’ should be written as, ‘to distinguish between different types’…
- L 395 (p 20) be careful how you phrase your suggestions about what future research ‘should do’, may I suggest instead writing, ‘It would be beneficial for future research to explore’…

Thank you for pointing us to this language use. We adopted reviewer’s suggestions.

- L 385-390 (p 20) pay attention to your run-on sentences.

We split these sentences into more sentences, so that the text is easier to follow (lines 457-462):

*“Future research may further investigate the relation between eating context and intentions to adopt personalised nutrition services by, for example, further specifying the different contextual factors that may affect an individual’s evaluation of personalised nutrition services. Moreover, future research could investigate how the enhancing effect of eating context on ambivalent feelings can be reduced, perhaps, by including contextual factors such as preferences or dietary requirements of family members in the personalised nutrition advice.”*

CONCLUSION:

- L 402 (p 21) may I suggest you remove the word ‘obviously’ as it is a polarising word and has the potential to make the reader look or feel stupid.
- L 403 (p 21) instead of writing ‘per person’, a more scientific way is to write ‘in individuals’…
- L 407 (p 21) instead of writing ‘someone’s eating context’, a more scientific expression would be, ‘taking individuals’ eating context’…

Thank you for pointing us to this language use. We adopted reviewer’s suggestions.

I am finished with my comments, but I do have a few questions: How can a policy or an incentive take into account an individual’s eating context? What is an example of what a policy should employ? Or what incentive should be employed? If you can list examples of some ideas you may have, then it offers a clearer picture of what can be done to improve areas that may need improving, in terms of trying to improve public health or personal health.

Thank you for your questions, these helped us to improve our manuscript. We extended the final section substantially (which we now labelled as ‘Conclusions and practical implications’)

Also, what specifically does personalise nutrition advice based on DNA profiling mean? What is required and how can individuals, who may be interested in participating, be assured that their DNA profiling will not be abused or used by governments who may desire to gather ‘profiles’ of certain people to use for whatever they may want to use DNA profiling for. Or companies that may try targeting susceptible profiles, how are these individuals protected. What sort of data protection is there for these personalised nutrition services?

We thank the reviewer for his or her interest in the topic. He/she asks many questions related to personalised nutrition based on DNA testing. We think these questions are largely beyond the scope of the current article since our study was not specifically focused on personalised nutrition advice based on DNA profiling. Nevertheless, in the practical implications we take some of these data protection aspects into account (see also previous comment) (lines 489-500):

*“[...], personalised nutrition services should take privacy issues seriously and communicate transparently about how personal data are protected. Similarly, providers of personalised nutrition should disclose their intentions for customer data use. For example, individuals who may be interested in participating in a personalised nutrition program should be informed that their profiles will not be shared with third parties who may use this information for commercial reasons. Security and anonymity of data should be made transparent to customers of personalised nutrition services, who should have the opportunity to provide informed consent. Additionally, an individual’s data or measures should only be collected if there is reliable evidence that personalising advice based on this information could influence health or deliver functional benefits (Adams et al., 2019). There should also be transparent communication as to how each piece of the collected information contributes to these (health) benefits.”*

Furthermore, Adams et al. (2019) argues that the potential contribution of personalised nutrition based on DNA profiling should not be overstated: “*For example, with some exceptions, the potential of individual genes as the basis of a PN approach to affect health is relatively small*.”

Personally, I find it a little scary that your research was funded by an agriculture and food company because that suggests they are interested in profiling people, which by default, I do not trust food and agricultural companies.

Our research is not funded by an agriculture and food company, but by the Dutch Top Sector Agri & Food. The Top Sector Agri & Food is one of the 9 top sectors in which The Netherlands excels worldwide. Businesses, universities, research centres, governments and social organisations work together in public-private partnerships on knowledge and innovation. See also the website: <https://topsectoragrifood.nl/en/> (we added this information to the Acknowledgements).

That said, although the research is indeed partly funded by private actors, you cannot say that our research is only funded by food and agricultural companies, since it is also co-financed by the Dutch government and universities and research institutes contribute to the Topsector’s research agenda with independent research.
