# Supplementary material for: Consumer acceptance of personalised nutrition: The role of ambivalent feelings and eating context
Source: PLoS One. 2020 Apr 13;15(4):e0231342. doi: 10.1371/journal.pone.0231342 (PMC7153894; doi:10.1371/journal.pone.0231342)
Supplement: Attachment — Submitted filename: PLOS ONE Second review of Consumer Acceptance.docx [file pone.0231342.s006.docx]

PLOS ONE Second review of:

CONSUMER ACCEPTANCE OF PERSONALISED NUTRITION: THE ROLE OF AMBIVALENT FEELINGS AND EATING CONTEXT

L = Line, p = page

Summary of the rewrite: The researchers took onboard the editors’ feedback, and the outcome has produced a much stronger article. The way in which the rewrite has been done is much better than the original manuscript. The English is better, there is more use of scientific language, which helps better with a logical flow of what the research problem was, what was done and what was found. The inclusion of both the Dutch and English questionnaire serves to strengthen the work and understand better what was done. I have only a few more suggestions which some are nit-picking but may be helpful. And some are questions which the authors might want to consider.

Question 1: Did the authors aim to try to validate this questionnaire? The authors discuss ‘pre-tested versions’ (L 161, p 9), but did they do a re-test. As the authors are probably aware, a re-test of the questionnaire (namely within 2 to 4 weeks) helps to validate the questionnaire, but it was not stated if this was the case.

Also, just a note for future studies when using a questionnaire: In the ‘Instruction’ portion of the questionnaire, it is best to guide the volunteer to answer the questions based on some period of time, such as the past 4 weeks, or the past year, otherwise it can lead to ambiguity in the findings.

ABSTRACT:

L 24: An ‘a’ is missing between “perceived as” and “barrier”.

L 25-29 Is a very long sentence and leads to confusion. ‘…the current study suggests the additional value of ambivalent feelings as an affective construct and eating context as a possible barrier’ are these two separate aspects? Or are they two aspects that combine as one problem? It might be better expressed if you separated them with a comma, ex: ‘the current study suggests the additional value of ambivalent feelings as an affective construct, and eating context as a possible barrier’… this helps to identify each as two separate aspects, that is, if that is what they are. Also, L 27 would be better to start a new sentence: ‘This implies that’…

L 54 (p 3) apologies for nit-picking here, but technically, when sentences contain more than two ‘ands’ it is best to try to group sentences that are connected with ‘and’. A sentence that can almost stand alone, but is still connected with and, should have a comma after it. It helps the sentence to flow better and yet stay connected for the reader. In other words: ‘These developments and technologies enable personal data collection and nutrition delivery services, and practitioners are beginning to use these tools’ …

L 67 (p 4) this ‘benefits and risks’ trade-off (should be in brackets just to make it clearer, crisper looking.

L 73 (p 4) instead of ‘receiving’ it should be ‘received’.

L 95 (p 5) because you use ‘in addition’ or ‘additionally’ quite often, you may want to begin this paragraph with ‘Moreover’ just to keep the readers interest.

L 111 (p 6) I like how you placed in brackets (as captured by the concept of risk-benefit calculus), that helps to emphasise your point. Well done.

L 127-130 (p 6) I am confused here. Were your 797 participants individuals who had never used ‘personalised nutrition services’ before? If this is the case, how can the questionnaire accurately measure the various aspects/constructs it’s trying to measure? Such as, qu. 3 Ambivalent Feelings “When I **would use** personalised nutrition services”… how can a volunteer who has never before used personalised nutrition services accurately answer this question? The same issue applies to question 4 (Personalisation Benefit and Privacy Risks) how can a volunteer, who hasn’t used these services before, answer questions relating to issues (based on past experience) on personalised nutrition services? That goes for any other questions (i.e. Q 7 and 9) that relates to usage of personalised nutrition services. Are you asking the volunteers to project (to try and answer) these questions? In addition, it now makes it unclear who your target audience is. Your Methods section does not make clear who your target audience is.

L 237 (p 14) ‘Participants provided’… the use of the term ‘provided’ suggests that the participants are actively giving you or handing you something, so ‘provided’ is not the correct term. Instead, you could just write, ‘Participants had’… Alternatively, you could write, ‘The construct with the highest mean score was found to be in *Personalisation Benefit* (…)’

L 237 ‘highest mean average’ is redundant, it should just be expressed as ‘the highest mean score’

L 237, ‘score to’ is not correct, it should be written ‘score in’…

L 252 (p 14) The authors write that ‘moderately to strongly correlated with all variables except *Eating Context Barrier*.’ However, I would like to point out that the correlation with ‘*Privacy Risk*’ of -0.13 is not considered moderate, but either weak or very weak; and the correlation of -0.31 can also be considered weak, depending on who you reference. Evans (1996) correlations state that: very weak: 0.00-0.19; weak: 0.20-0.39; moderate: 0.40-0.59; strong: 0.60-0.79; very strong: 0.80-1.00. And Cohen (1988) correlations state that: weak: 0.1-0.3; moderate: 0.3-0.5; strong: >0.5.

[Cohen, J., 1988. Statistical power analysis for the behavioural sciences (2^nd^ Ed). Erlbaum: Hillside, NJ. Evans, J.D., 1996. Straightforward statistics for the behavioural sciences. Thomson Brooks/Cole Publishing: Pacific Grove, Calif.]

L 325 (p 18) …’context is more perceived as’ … should be written as ‘context is perceived more as’…

L 374 (p 20) ‘few studies on personalised nutrition have looked at…’ it is more scientific to express it as ‘few studies… have explored’… or ‘few studies… have investigated’…

L 376 (p 20) the word ‘suggest’ should have an s at the end, so it should be ‘suggests’…

L 378 (p 20) ‘This suggests that a person’s eating context’… the more scientific way to express it is: ‘This suggests that an individual’s eating…’

L 390 (p 21) …’which in turn negatively affect’… I believe there should be an ‘s’ added to the word ‘affects’…

L 390 (p 21) ‘Further research is needed to further elucidate’… it might be better to state that ‘More research is needed to further elucidate’, as it flows better. In the English language, which I can appreciate is not your Mother tongue, you keep the reader’s attention by trying to use a variety of words, instead of the same word constantly or more than once.

L 433 (p 22) ‘variety of more specific (fictional) advices’… The word advice is both singular and plural. Perhaps a better way to express this sentence and maintain what you are trying to say, you could write: ‘variety of more specific types of (fictional) advice’ …

Also a note for your 4.2 Limitations sections, if the questionnaire has not been validated, this needs to be considered a limitation. Validating the questionnaire is a way of measuring the consistency of how individuals are answering the questions at any given time.
