# Supplementary material for: Consumer acceptance of personalised nutrition: The role of ambivalent feelings and eating context
Source: PLoS One. 2020 Apr 13;15(4):e0231342. doi: 10.1371/journal.pone.0231342 (PMC7153894; doi:10.1371/journal.pone.0231342)
Supplement: Attachment — Submitted filename: PONE-D-19-26890R1_Rebuttal revisionV1.docx [file pone.0231342.s007.docx]

Ms. Ref. No.: PONE-D-19-26890R1

Title: Consumer acceptance of personalised nutrition: The role of ambivalent feelings and eating context

Journal: PLOS ONE

# EDITOR'S comments/ Reviewers' comments:

Thank you very much again for the constructive and helpful review of the manuscript and for inviting us to submit a further revised version of the manuscript. We revised the manuscript based on the comments. Both a marked-up copy of the manuscript with tracked changes as well as an unmarked version are resubmitted to the journal.

## Reviewer #1:

The authors should be commended on the care taken to address all the comments. I am happy that my comments have been addressed.

Thank you for your kind words on the paper. We are glad to hear that all your comments are satisfactorily addressed.

Whilst there is some duplication of means and SD between tables 2 and 3, I can see why the authors have included this.

Yes, there is indeed duplication of means and SD between Tables 2 and 3. Tables 2 presents the constructs and its underlying measurement items. In Table 2 also the overall construct means and SD are presented, which is also the case in Table 3. However, in this latter table, also the intercorrelations between the constructs are displayed.

On reading the paper, I did notice a few minor corrections required to English:

• Line 73 – should be ‘received’ instead of ‘receiving’

• Line 124 – should be ‘To ensure a sample that was nationally representative’ instead of ‘is’.

• Line 161 – should read ‘Before data collection started’.

• Line 376 – should read ‘suggests that’ instead of ‘suggest that’

• Line 433 – should be ‘advice’ instead of ‘advices’.

Thank you, we corrected these errors in English language use.

## Reviewer #2:

The researchers took onboard the editors’ feedback, and the outcome has produced a much stronger article. The way in which the rewrite has been done is much better than the original manuscript. The English is better, there is more use of scientific language, which helps better with a logical flow of what the research problem was, what was done and what was found. The inclusion of both the Dutch and English questionnaire serves to strengthen the work and understand better what was done. I have only a few more suggestions which some are nit-picking but may be helpful. And some are questions which the authors might want to consider.

We are happy to hear that all comments and feedback were satisfactorily addressed and that our article improved a lot. We would also like to thank the reviewer very much for his/her helpful feedback to further improve the paper.

Question 1: Did the authors aim to try to validate this questionnaire? The authors discuss ‘pre-tested versions’ (L 161, p 9), but did they do a re-test. As the authors are probably aware, a re-test of the questionnaire (namely within 2 to 4 weeks) helps to validate the questionnaire, but it was not stated if this was the case.

We indeed did not conduct a re-test*, but as can be read in the Measures-section (2.2), existing, validated measurement scales were used to measure the different constructs in our study. The questionnaire was further validated by testing the reliability and validity of the measurement scales. As a measure of scale (composite) reliability, we used Cronbach’s alpha to test the internal consistency of the measurement items of the constructs, that is, how closely related a set of items are as a group. Table 2 of the manuscript shows that Cronbach’s alpha was higher than .80 for all constructs, indicating good reliability. In addition, to measure the validity of the constructs, we looked at convergent and discriminant validity. Convergent validity refers to the degree to which two measurement items that theoretically should be related, are factually related. Except for looking at the inter-item correlations between the individual items of a scale (which should be greater than .50), criteria for convergent validity require the Average Variance Extracted (AVE) greater than 0.5, standardized factor loading of all items not less than 0.5, and composite reliability not less than 0.7 (Hair et al., 2010). As can be seen in Table 2, the variables that were used have high factor loadings (> .50), high composite reliabilities (Cronbach's alphas >0.7) and AVE greater than 0.50, which indicates convergent validity. Additionally, discriminant validity tests whether concepts or measurements that are not supposed to be related are actually unrelated. Discriminant validity can be tested by checking whether the square root of the AVE for each construct is greater than its correlations with the other constructs (see Fornell and Larcker, 1981). When looking at Tables 2 and 3 we can conclude that this was indeed the case for our constructs, indicating that all factors in the measurement model possess good discriminant validity.

We agree that, although this information implicitly was already present in the manuscript (namely shown in Tables 2 and 3), this could have been better described. So, we now added the information described above to the manuscript (see lines 198-216).

*) Note that we incorporate this as a limitation in the revised version of the paper. See also our response to the final comment of the reviewer.

Also, just a note for future studies when using a questionnaire: In the ‘Instruction’ portion of the questionnaire, it is best to guide the volunteer to answer the questions based on some period of time, such as the past 4 weeks, or the past year, otherwise it can lead to ambiguity in the findings.

We thank the reviewer for this advice. Although in the case of our study, instructing participants to think about a past period of time is not applicable because we worked with a hypothetical scenario, it could have nevertheless been helpful to guide participants to answer the questions based on a concrete situation where the use of personalised nutrition may be relevant. Think of a health problem or a specific dietary request. We added this as a suggestion for future studies in the ‘Limitations and future research’ section (Section 4.2), lines 456-459: “*Additionally, to further reduce ambiguity in the findings, it could be helpful to guide participants, for example in the ‘Introduction’ part of the questionnaire, to answer the questions based on a concrete situation where the use of personalised nutrition may be relevant.*”

- L 24: An ‘a’ is missing between “perceived as” and “barrier”.

Thank you. We corrected this.

- L 25-29 Is a very long sentence and leads to confusion. ‘…the current study suggests the additional value of ambivalent feelings as an affective construct and eating context as a possible barrier’ are these two separate aspects? Or are they two aspects that combine as one problem? It might be better expressed if you separated them with a comma, ex: ‘the current study suggests the additional value of ambivalent feelings as an affective construct, and eating context as a possible barrier’… this helps to identify each as two separate aspects, that is, if that is what they are. Also, L 27 would be better to start a new sentence: ‘This implies that’…

We thank the reviewer for these suggestions. We now separated the two aspects with a comma, as these indeed are two separate aspects. And we divided the long sentence in two separate sentences to make them more readable.

- L 54 (p 3) apologies for nit-picking here, but technically, when sentences contain more than two ‘ands’ it is best to try to group sentences that are connected with ‘and’. A sentence that can almost stand alone, but is still connected with and, should have a comma after it. It helps the sentence to flow better and yet stay connected for the reader. In other words: ‘These developments and technologies enable personal data collection and nutrition delivery services, and practitioners are beginning to use these tools’ …

Thank you, we improved this sentence with adding an extra comma.

- L 67 (p 4) this ‘benefits and risks’ trade-off (should be in brackets just to make it clearer, crisper looking.

Thank you, we put this in brackets.

- L 73 (p 4) instead of ‘receiving’ it should be ‘received’.

Thank you, we corrected this error.

- L 95 (p 5) because you use ‘in addition’ or ‘additionally’ quite often, you may want to begin this paragraph with ‘Moreover’ just to keep the readers interest.

Thank you, we incorporated this suggestion.

- L 111 (p 6) I like how you placed in brackets (as captured by the concept of risk-benefit calculus), that helps to emphasise your point. Well done.

Thank you for the compliment!

- L 127-130 (p 6) I am confused here. Were your 797 participants individuals who had never used ‘personalised nutrition services’ before? If this is the case, how can the questionnaire accurately measure the various aspects/constructs it’s trying to measure? Such as, qu. 3 Ambivalent Feelings “When I would use personalised nutrition services”… how can a volunteer who has never before used personalised nutrition services accurately answer this question? The same issue applies to question 4 (Personalisation Benefit and Privacy Risks) how can a volunteer, who hasn’t used these services before, answer questions relating to issues (based on past experience) on personalised nutrition services? That goes for any other questions (i.e. Q 7 and 9) that relates to usage of personalised nutrition services. Are you asking the volunteers to project (to try and answer) these questions? In addition, it now makes it unclear who your target audience is. Your Methods section does not make clear who your target audience is.

The reviewer is right, the 797 participants were indeed individuals who had never used ‘personalised nutrition services’ before. The target group of our study are people who are not experienced with personalised nutrition services, as we want to gain more insight in the acceptance potential of personalised nutrition services. To make things more clear we added the following to the relevant sentence (added clause in italics) (lines 127-133): “To avoid bias in the results that may be caused by including people who currently use personalised nutrition advice—and who may see its benefits and risks differently, based on their experiences—an additional 40 participants, *who have experience with personalised nutrition services*, were omitted from the analyses. *The target group of our study are people who are not experienced with personalised nutrition services, as we want to gain more insight in the acceptance potential of personalised nutrition services.*”

We agree that the questions are hypothetical and that participants who have never used personalised nutrition services before cannot accurately answer the questions. At least not based on their experience. However, based on the provided definition, they could have developed expectations regarding the different aspects of personalised nutrition. This type of questioning is not unusual in research looking at the adoption potential of new products or services. For example, these type of questions are also applied in studies that we cited in our paper (see for example: Berezowska et al., 2015; Poinhos et al., 2014).

Note that starting the questionnaire with a definition not only has its shortcomings (where we already elaborated on in the discussion), but simultaneously has the advantage that it gives participants an idea of what they should think about when it comes to personalised nutrition, thereby preventing that participants all interpret the questions based on their own idea of what personalised nutrition is about.

- L 237 (p 14) ‘Participants provided’… the use of the term ‘provided’ suggests that the participants are actively giving you or handing you something, so ‘provided’ is not the correct term. Instead, you could just write, ‘Participants had’… Alternatively, you could write, ‘The construct with the highest mean score was found to be in Personalisation Benefit (…)’
- L 237 ‘highest mean average’ is redundant, it should just be expressed as ‘the highest mean score’
- L 237, ‘score to’ is not correct, it should be written ‘score in’…

Thank you, we addressed these language issues and now re-worded the sentence into: ‘The construct with the highest mean score was found to be in Personalisation Benefit (…)’

- L 252 (p 14) The authors write that ‘moderately to strongly correlated with all variables except Eating Context Barrier.’ However, I would like to point out that the correlation with ‘Privacy Risk’ of -0.13 is not considered moderate, but either weak or very weak; and the correlation of -0.31 can also be considered weak, depending on who you reference. Evans (1996) correlations state that: very weak: 0.00-0.19; weak: 0.20-0.39; moderate: 0.40-0.59; strong: 0.60-0.79; very strong: 0.80-1.00. And Cohen (1988) correlations state that: weak: 0.1-0.3; moderate: 0.3-0.5; strong: >0.5.
- [Cohen, J., 1988. Statistical power analysis for the behavioural sciences (2nd Ed). Erlbaum: Hillside, NJ. Evans, J.D., 1996. Straightforward statistics for the behavioural sciences. Thomson Brooks/Cole Publishing: Pacific Grove, Calif.]

We agree with the reviewer that a correlation of -0.13 cannot be considered moderate. This was a little careless mistake from our side. We corrected this and also added Cohen as a reference in the referred sentence.

- L 325 (p 18) …’context is more perceived as’ … should be written as ‘context is perceived more as’…

Corrected.

- L 374 (p 20) ‘few studies on personalised nutrition have looked at…’ it is more scientific to express it as ‘few studies… have explored’… or ‘few studies… have investigated’…

Thank you, we corrected this.

- L 376 (p 20) the word ‘suggest’ should have an s at the end, so it should be ‘suggests’…

Corrected.

- L 378 (p 20) ‘This suggests that a person’s eating context’… the more scientific way to express it is: ‘This suggests that an individual’s eating…’

Thank you, we adapted this.

- L 390 (p 21) …’which in turn negatively affect’… I believe there should be an ‘s’ added to the word ‘affects’…

The word ‘affect’ refers to ‘ambivalent feelings’, so it should indeed be plural. However, we agree that the sentence was not completely correct, as there was stated ‘while it does positively affect ambivalent feelings’, but ‘it’ should be ‘they’ as it refers to ‘eating context barriers’, which is also plural. In sum, we adapted the sentence as follows: “We expect this with greater reason, because eating context barriers show no significant inter-correlation with intention to use personalised nutrition advice, while they do positively affect ambivalent feelings, which in turn negatively affect intention to use personalised nutrition advice.”

- L 390 (p 21) ‘Further research is needed to further elucidate’… it might be better to state that ‘More research is needed to further elucidate’, as it flows better. In the English language, which I can appreciate is not your Mother tongue, you keep the reader’s attention by trying to use a variety of words, instead of the same word constantly or more than once.

Thank you, we adapted this.

- L 433 (p 22) ‘variety of more specific (fictional) advices’… The word advice is both singular and plural. Perhaps a better way to express this sentence and maintain what you are trying to say, you could write: ‘variety of more specific types of (fictional) advice’ …

Thank you for the suggestion, we adapted this.

- Also a note for your 4.2 Limitations sections, if the questionnaire has not been validated, this needs to be considered a limitation. Validating the questionnaire is a way of measuring the consistency of how individuals are answering the questions at any given time.

Although we validated the questionnaire in the sense that we checked for reliability and validity for the used measurement scales (see our response to one of the previous comments), we indeed did not measure the consistency of how individuals are answering the questions at any given time. We added this as limitation and recommendation for future research (lines 465-467): *“[...] to further test the consistency of the used measurement scales (i.e., how individuals are answering to the used measures at any given time), future research may consider a longitudinal data collection, which may also be used to assess test-retest reliability.”*
